# Wrinkle-Assisted Nanofluidic Memristors for Geometry-Dependent Ionic Memory

**DOI:** 10.1021/acsnano.5c20258

**Published:** 2026-04-03

**Authors:** Minsu Kwon, Dongwoo Seo, Taesung Kim

**Affiliations:** † Department of Mechanical Engineering, 131639 Ulsan National Institute of Science and Technology (UNIST), 50 UNIST-Gil, Ulsan 44919, Republic of Korea; ‡ Department of Biomedical Engineering, Ulsan National Institute of Science and Technology (UNIST), 50 UNIST-Gil, Ulsan 44919, Republic of Korea

**Keywords:** micro-/nanofluidics, micro-/nanofabrication, wrinkle lithography, wrinkle-based nanochannels, nanofluidic memristor, geometry-dependent tunable ionic memory

## Abstract

Electronic memristors have greatly advanced artificial synapse research, but their reliance on electron transport, which differs intrinsically from the ion-mediated signaling and spatiotemporal dynamics of biological synapses. Here, we present wrinkle-based, geometry-tunable nanochannels integrated within a hybrid polydimethylsiloxane (PDMS)-OSTEMER chip as a simple, low-cost, and reproducible platform for ionic memory. Exploiting the modulus mismatch between PDMS and OSTEMER, nanoscale wrinkles were selectively preserved only within the designated bridge region, forming a controllable array of nanochannels that govern ionic transport. By tailoring the number and length of these nanochannels, ionic conduction and memory characteristics could be precisely modulated. The resulting wrinkle-based nanochannel array device (WNAD) exhibited pronounced memristive hysteresis and effectively emulated key synaptic plasticity behaviors, including short-term plasticity (STP), paired-pulse facilitation (PPF), and reproducible potentiation-depression cycles. Moreover, the WNAD reproduced cumulative reinforcement under repeated stimulation, demonstrating geometry-dependent memory consolidation analogous to biological conditioning. Collectively, this study established wrinkle-based nanochannels as a bioinspired nanofluidic platform for ionic memory, bridging confined ionic transport and neuromorphic functionality.

## Graphical abstract

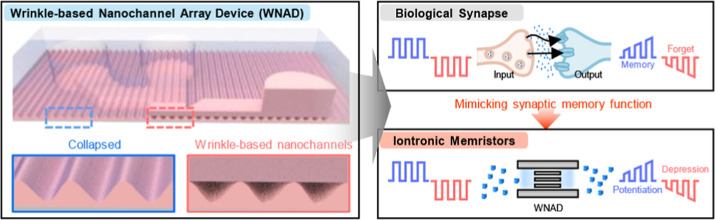

## Introduction

The conventional computing paradigm, based on the von Neumann architecture, has enabled modern electronics for decades but remains fundamentally constrained by the physical separation of memory and processing units.^1^ This separation, commonly referred to as the von Neumann bottleneck, limits parallel information flow, increases energy consumption, and hinders adaptive, real-time information processing.^2,3^ In contrast, biological neural systems integrate memory and computation within neurons and synapses, enabling massively parallel and energy-efficient signal processing.^4^ Inspired by these characteristics, neuromorphic computing aims to emulate artificial synaptic elements capable of exhibiting the adaptive behavior of neural networks.^5,6^ Among various approaches, memristors have emerged as key building blocks for neuromorphic systems because their conductance can be continuously modulated by the history of stimuli, mimicking synaptic weight evolution.^7,8^ Electronic memristors, in particular, have achieved significant milestones^9^ in device integration, switching speed, and scalability, but their operation relies on electronic charge transport and defect-mediated processes, which intrinsically differ from the ion-mediated signaling and spatiotemporal dynamics of biological synapses. By contrast, iontronic devices exploit ionic transport within confined channels, closely resembling neurotransmitter-mediated signal transmission in biological systems.^10^ Because synaptic communication in living organisms fundamentally relies on the migration, accumulation, and relaxation of ions, ion-based platforms provide a more biomimetic framework for implementing synaptic functions. Such systems can naturally reproduce key aspects of synaptic plasticity including short-term plasticity (STP), paired-pulse facilitation (PPF), post-tetanic potentiation (PTP), and persistent conductance modulation,^11,12^ making iontronic memristors a promising platform for neuromorphic signal processing.

To achieve such synaptic behaviors, iontronic memristors have been implemented using various materials incorporating nanostructures such as nanopores and nanochannels.^12–14^ Under nanoconfinement, ionic transport becomes governed by surface charge, leading to selective ionic transport within confined channels. This surface-governed transport results in ionic accumulation and depletion, manifested as ion concentration polarization (ICP). Importantly, these history-dependent ionic redistribution processes underpin the nonlinear transport and memristive behavior observed in nanofluidic systems.^6,13,15^ However, realizing nanoscale confinement for iontronic memristors generally relies on two main fabrication strategies. One approach employs top-down nanofabrication techniques, such as electron-beam lithography of focused ion beam milling, to define nanopores and nanochannels with high precision and reproducibility. Despite their accuracy, these methods require extensive resources and remain costly and low-throughput.^16,17^ Alternatively, bottom-up strategies based on 2D or 3D materials, including graphene oxide, MXenes, molybdenum disulfide, and hydrogels, have been explored to achieve ionic selectivity. While such material-based approaches offer atomic-scale confinement, they often face challenges in large-area integration, active control of ionic pathways, and mechanical stability, limiting the reproducibility and tunability of ionic transport. Mechanically induced wrinkling has emerged as an unconventional yet simple, rapid, and low-cost nanofabrication method capable of generating periodic nanoscale grooves with tunable dimensions.^18–20^ When appropriately sealed, these wrinkle-induced grooves can be transformed into enclosed nanochannels whose number and length are directly programmable through macroscopic design parameters. This mechanically defined nanoconfinement enables reliable access, reproducibility, and precise geometric control of ionic transport without relying on complex top-down nanofabrication or material-intrinsic pore formation. As such, wrinkle-based nanochannels provide a scalable and accessible structural platform for iontronic devices, including ionic memristors.

In this work, we introduced a hybrid PDMS/OSTEMER-based wrinkle nanochannel array device (WNAD) that integrates a microfluidic channel network on a wrinkle-patterned substrate. By deliberately designing OSTEMER–OSTEMER or PDMS-OSTEMER interfaces, wrinkle-induced grooves were selectively preserved only within a designated bridge channel connecting two main channels, thereby confining ionic transport exclusively through the wrinkle-based nanochannels.^21,22^ Systematic characterization demonstrated that both the number and length of the nanochannels are directly programmable through the bridge channel geometry, enabling precise and reproducible control of ionic transport behavior. The device exhibited voltage-dependent ion accumulation-depletion dynamics, leading to geometry-dependent memristive hysteresis and synaptic behaviors analogous to STP. Importantly, the memristive characteristics could be engineered simply by adjusting the bridge channel geometry, without additional nanofabrication steps. Together, the WNAD established a mechanically programmable nanofluidic platform that links controlled ionic transport with neuromorphic iontronic functionality, providing a practical route toward scalable and reproducible artificial synaptic devices.^23^

## Results

### Concept of the WNAD

Figure 1A presents both the cross-sectional and top views of the WNAD integrating wrinkle-based nanochannels. The cross-sectional view shows how the wrinkle surface, embedded within the PDMS/OSTEMER chip, forms nanochannels that serve as ionic transport pathways. The top view shows the spatial arrangement of the injection (10 μm deep), bridge (10 μm deep), and main channels (25 μm deep), providing an integrated perspective on how wrinkle nanochannels are positioned and interconnected within the WNAD. Figure 1B schematically illustrates parallel, multiple, and tunable wrinkle nanochannels generated by spontaneous surface wrinkling, which yields periodic nanoscale grooves that laterally confine ions between two main channels.^18^ This nanoscale confinement produces selective ionic transport under an applied electric field, establishing the fundamental mechanism for memristive behavior. Figure 1C depicts the fabrication of the wrinkle surface via soft lithography. Wrinkles were first induced by bending a PDMS/Poly­(vinyl alcohol) (PVA) bilayer (i) and subsequently transferred onto a NOA63-coated PET substrate (ii). A PDMS master mold was replicated from the wrinkled NOA63 surface (iii), followed by the transfer of wrinkle patterns into OSTEMER through UV nanoimprinting (iv). This additional transfer step into OSTEMER was critical because the reactive functional groups within OSTEMER (e.g., thiols, epoxides, hydroxyls) enabled covalent bonding between wrinkle interfaces, yielding mechanically robust and chemically durable nanochannel structures compared to those formed via NOA63 alone.^24,25^ Detailed procedures are described in Part I in Supplementary Notes and Figure S1 in Supplementary Figures.

**Figure 1:**
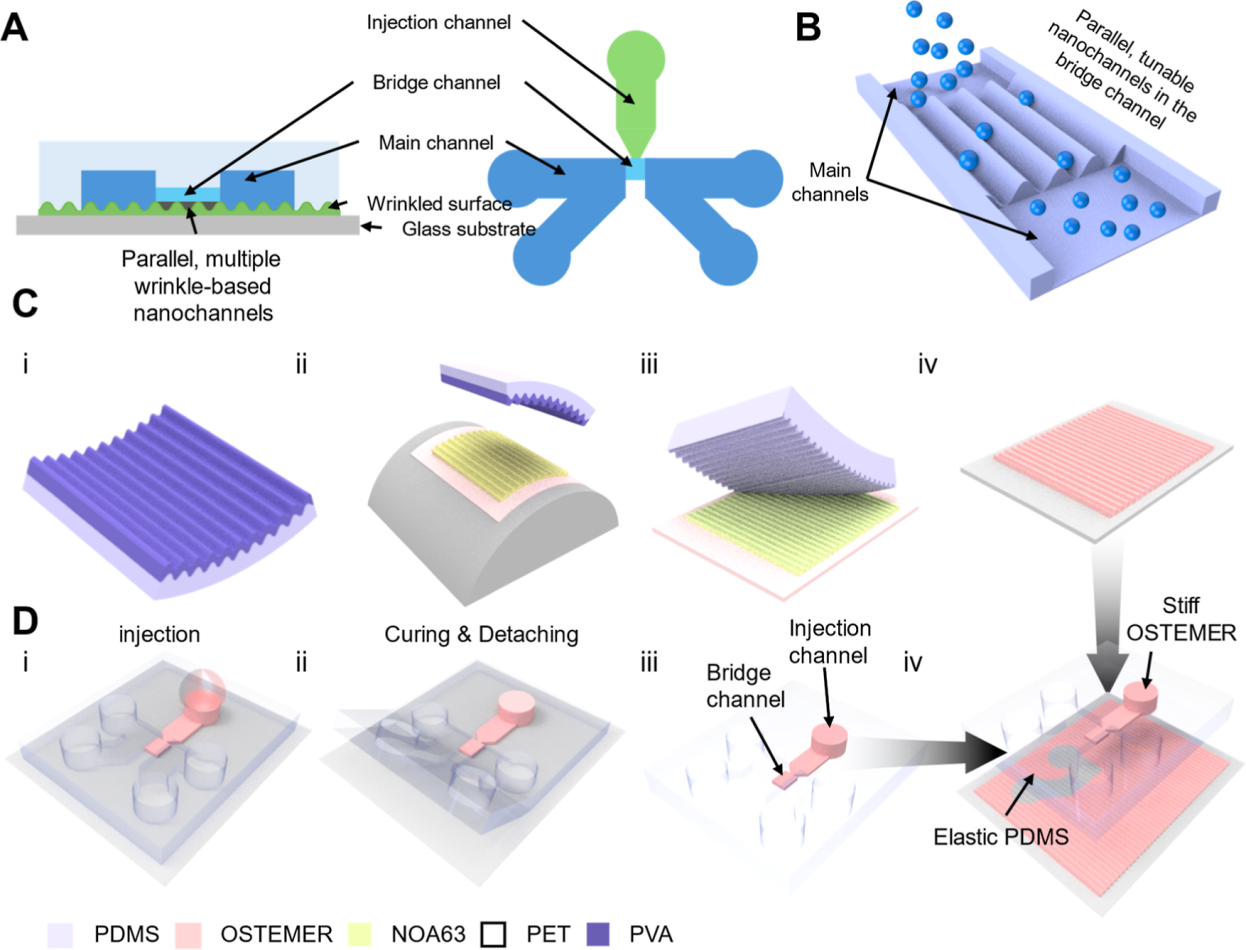
Fabrication process and schematic illustration of a wrinkle-based nanochannel array device (WNAD). (A) Cross-sectional (left) and top (right) views of the WNAD. Wrinkles underneath the two microchannels were tightly sealed, while those underneath the injected OSTEMER in the bridge channel remain intact, providing nanofluidic pathways for ions. (B) Ionic transport via wrinkle-based nanochannels. The number of the parallel wrinkle nanochannels was manipulated by adjusting the geometry of the bridge channel, forming nanoscale grooves that confine ionic flow and induce nonlinear transport leading to a memristive *I*–*V* curve. (C) Fabrication of a wrinkle-based nanochannel array substrate. Wrinkled patterns were formed in PDMS/PVA (i), transferred to NOA63 (ii), and replicated in PDMS (iii), and OSTEMER (iv). (D) Fabrication of the PDMS/OSTEMER hybrid chip. The bridge channel between the two main channels was filled with OSTEMER resin using the capillary effect, followed by UV curing (i). The PDMS/OSTEMER hybrid chip was peeled off (ii) and then bonded with the nanochannel array substrate (iii), completing the WNAD (iv).

Figure 1D illustrates the fabrication of the WNAD. A PDMS microfluidic layer was first bonded to a silane-treated glass slide after mild O_2_ plasma treatment, followed by capillary-driven infusion of OSTEMER resin through the injection channel (i). The resin selectively entered the bridge channel due to surface tension-driven Laplace pressure,^26–28^ the smaller hydraulic diameter of the injection and bridge channels generated a sufficiently high Laplace pressure to advance the resin (ii), whereas the wider main channels exhibited a lower Laplace pressure that prevented spontaneous filling. As a result, the OSTEMER resin was confined within the bridge channel region and halted at the liquid–air interface at the junction with the main channels, enabling self-limited, localization-based filling. Detailed fabrication steps and wetting mechanisms are provided in Figures S2 and S3. After UV curing (365 nm, 300 mW, 20 s) and demolding, the PDMS/OSTEMER hybrid chip was obtained (iii). A subsequent O_2_ plasma treatment (100 sccm, 100 W) facilitated covalent bonding to the wrinkle-patterned substrate (iv). Strong interfacial adhesion was achieved through condensation between PDMS -Si–OH and OSTEMER –OH groups, forming −Si–O–C- linkages, in addition to thiol-epoxide as well as hydroxyl ring–opening reactions between OSTEMER layers. These combined reactions provided a chemically stable and mechanically integrated hybrid structure with firmly bonded wrinkle nanochannel interfaces.

### Selective Formation and Manipulation of Functional Nanochannels

Figure 2 illustrates the selective formation of wrinkle-based nanochannels by controlling the elastic modulus at the bonding interfaces. Figure 2A schematically shows the WNAD, in which wrinkle-based nanochannels are embedded within the bridge channel positioned between two main channels. Figure 2B presents the cross-sectional view along A*–*A′, illustrating the comparison between the PDMS-OSTEMER and OSTEMER–OSTEMER interfaces. At the PDMS-OSTEMER interface, the low Young’s modulus of PDMS enabled conformal deformation into the wrinkle grooves, leading to complete collapse of the nanoscale cavities and thus eliminating nanochannel formation. This conformal sealing was beneficial for preventing unintended ionic leakage outside the designed nanochannel region. In contrast, the OSTEMER–OSTEMER interface within the bridge channel retained the original wrinkle morphology due to the higher modulus of OSTEMER, thereby preserving well-defined nanochannels capable of supporting ionic transport. The SEM images in Figure 2C experimentally confirmed this modulus-dependent selective bonding behavior. In the PDMS-contact region, the wrinkle topography was mechanically flattened, resulting in the loss of nanoscale channels. Conversely, the wrinkle features in the OSTEMER–OSTEMER region remained intact, demonstrating that wrinkle-based nanochannels were only maintained where both interfacing layers possessed sufficient rigidity. Consequently, the bridge channel served as the sole active ionic transport pathway.

**Figure 2:**
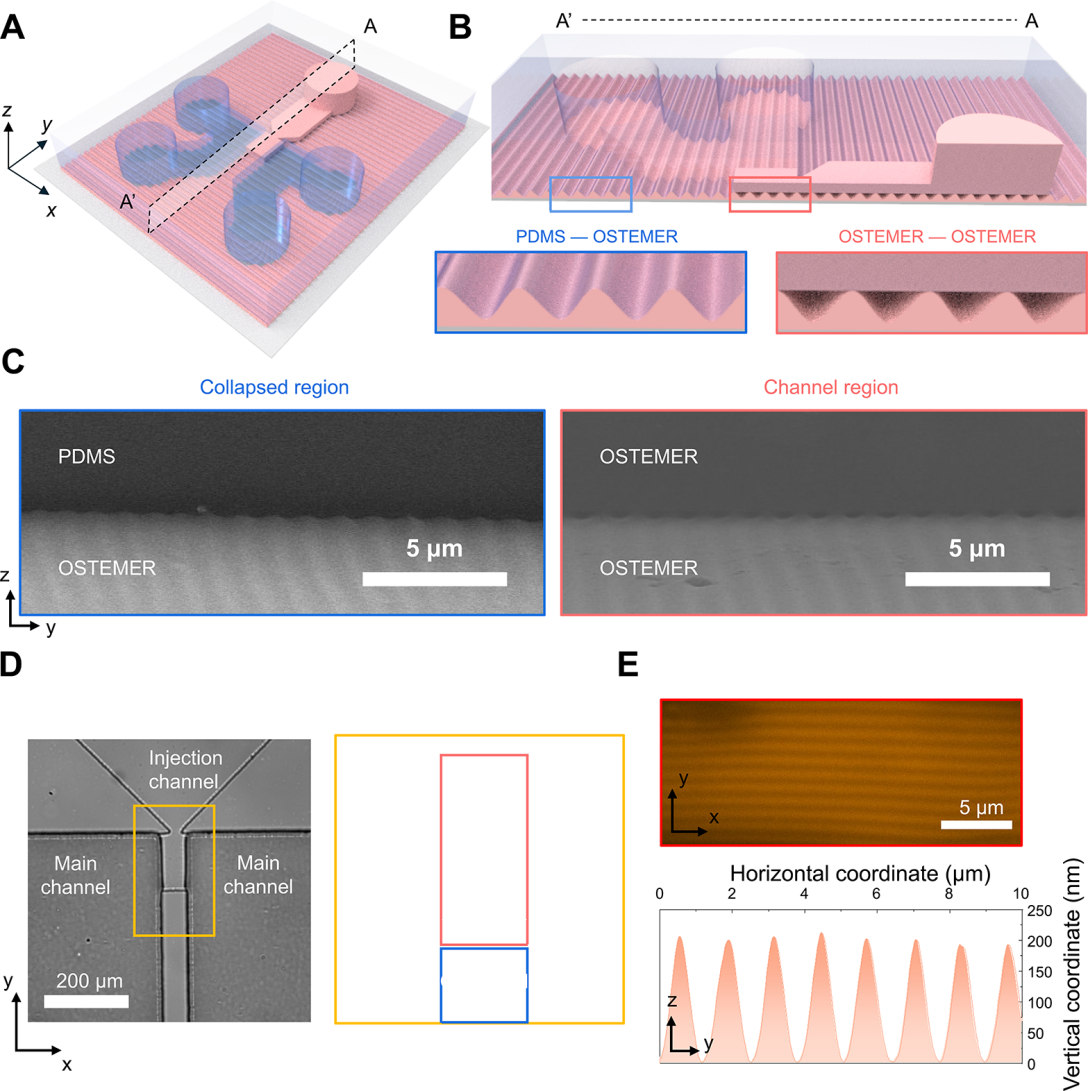
Illustration of the WNAD and its characterization. (A) Schematic overview of the WNAD consisting of the PDMS/OSTEMER top layer and the OSTEMER bottom substrate. (B) Schematic of the cross-section of the WNAD containing the selectively formed wrinkle-based nanochannels (red) and collapsed region (blue). (C) SEM images of the collapsed region and the nanochannel one. (D) Optical images of the WNAD in which wrinkle-based nanochannels connect the two main channels across the bridge channel. (E) Fluorescence image of the periodic wrinkle morphology across the bridge channel and AFM analysis of the wavelength and amplitude of the nanochannels.

Figure 2D shows top-view optical microscopy images of the WNAD. Both the injection and bridge channels were fabricated in OSTEMER, with the bridge channel acting as an array of nanochannels bridging the two main channels. The width (*w*) of the bridge channel was defined as *w* = *n*λ (*n* ∝ *w*), where *n* represents the number of nanochannels and λ denotes the wrinkle wavelength. The bridge channel length (*L*) was equivalent to the nanochannel length (*l*, i.e., *L* = *l*), allowing systematic tuning of both nanochannel number and length through bridge channel geometry design. Importantly, this nanochannel array was fabricated entirely via wrinkle-assisted nanofabrication, without relying on conventional high-resolution nanolithography. As shown in Figure 2E, fluorescence imaging confirmed the continuous filling of an ionic buffer solution through the wrinkle-based nanochannels in the bridge channel, validating their role as fluidic conduits. AFM characterization showed a wrinkle wavelength of approximately 1.1 μm and amplitude (*h*) of about 200 nm, consistent with the formation of confined nanochannels. Collectively, these results verify that functional nanochannels are selectively formed only at the OSTEMER–OSTEMER interface and demonstrate that their number and length can be precisely engineered by tailoring the bridge channel geometry.

### Geometry-dependent Hysteresis in *I*–*V* Characteristics

Figure 3 investigates how wrinkle-based nanochannels regulate ionic transport under applied electric fields and how this regulation gives rise to geometry-dependent hysteretic current responses. Figure 3A schematically illustrates ICP occurring at the junction between the main channels and wrinkle-based nanochannels.^15,29^ Note that the zeta potential of plasma-treated OSTEMER has been reported to approximately −38.5 mV,^30^ imparting negative surface charge to the nanochannel walls. At equilibrium, cations and anions are nearly uniformly distributed in the electrolyte, with a slight accumulation of cations inside the nanochannels due to electrostatic attraction. Upon applying an external bias, this equilibrium is distributed in a polarity-dependent manner. The forward bias induces cation depletion near the biased nanochannel entrance, whereas a reverse bias promotes cation accumulation at the same junction, with the spatial distribution inverted when the polarity is reversed. This accumulation-depletion transition generates asymmetric ionic distributions at the nanochannel junction, forming the physiochemical basis for the hysteretic *I*–*V* characteristics^31^ observed in the device. Dynamic ionic redistribution was experimentally visualized using fluorescein isothiocyanate (FITC) as a tracer. As shown in Figure S4, fluorescence images recorded at *t* = 0, 2, and 4 min under forward bias revealed tracer accumulation on one side of the nanochannels and depletion on the opposite side, while reversing the bias inverted this spatial distribution. These observations confirm that accumulation-depletion zones form reversibly within the wrinkle-based nanochannels and that their localization is governed by the direction of the applied electric field. Further details are provided in Part II in Supplementary Notes.

**Figure 3:**
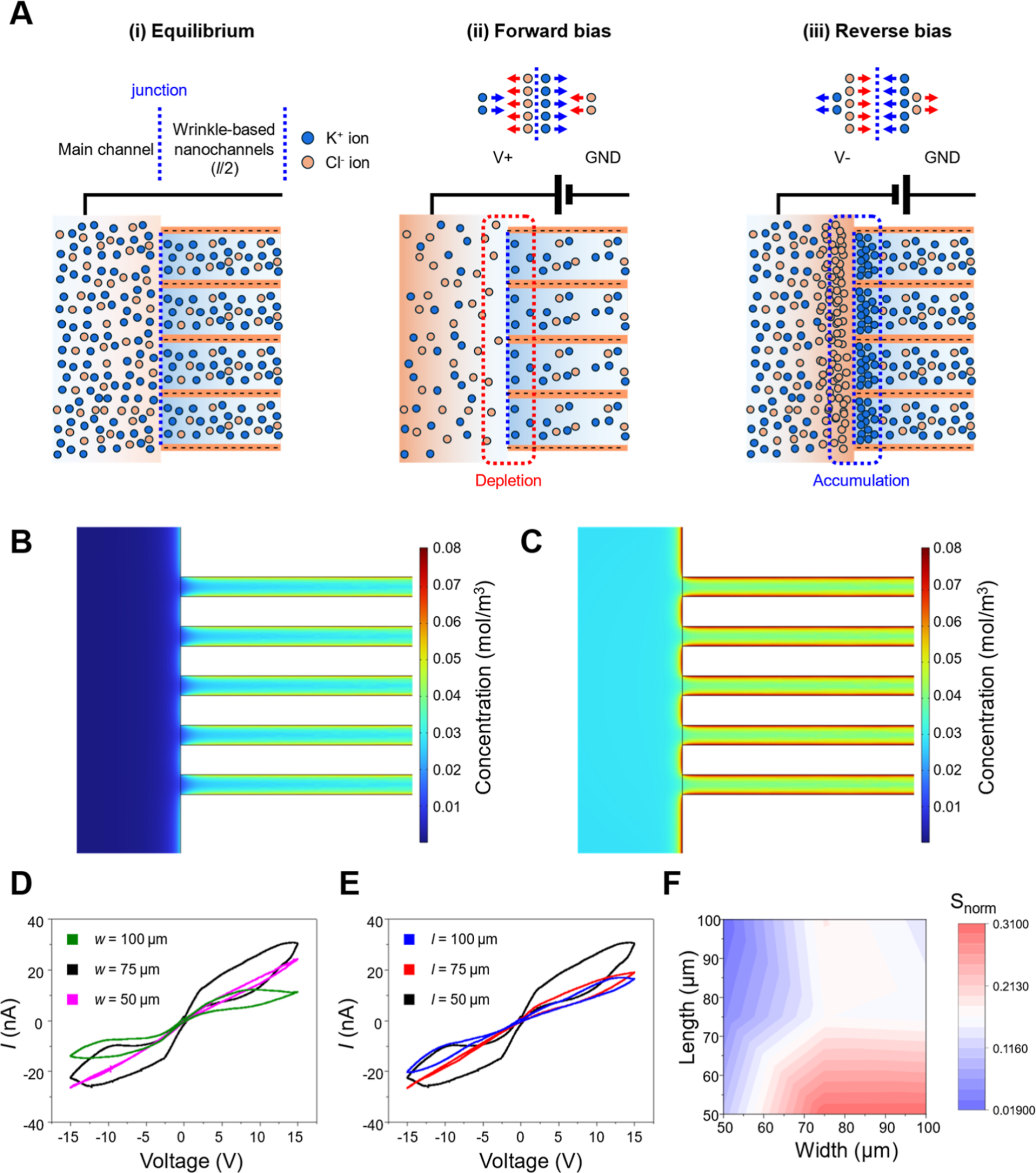
Ion distributions along the wrinkle-based nanochannels and their memristive behavior. (A) Schematic illustration of ionic distributions from the left main channel to the center of the wrinkle-based nanochannels (i.e., left–right symmetry). (i) Equilibrium. (ii) Forward bias forming an accumulation region, (iii) reverse bias forming a depletion region. (B,C) Numerical simulation of ionic concentration distribution near the left junction. (B) Forward bias showing a depletion region and (C) reverse bias showing an accumulation region. (D,E) Effects of the width and length of the bridge channel on the memristive hysteresis based on *I*–*V* measurements. (F) The normalized hysteresis loop areas (*S*_*n*_) and corresponding contour plot with respect to the width and length of the bridge channel.

Numerical simulations reproduced these asymmetric ionic behaviors (Figure 3B,C). For computational feasibility, the model was simplified to five parallel nanochannels. Under a +1 V bias, a pronounced K^+^ depletion zone formed at the nanochannel entrance, whereas a −1 V bias generated K^+^ accumulation. Despite the simplifications in the model, the results captured the essential features of the system, including cation selectivity arising from negatively charged walls and overlapping electric double layers (EDLs). For both bias polarities, the cation concentration inside the nanochannels consistently exceeded that of anions. During linear sweep voltammetry (LSV), incomplete relaxation of accumulated/depleted ions resulted in a temporal lag between forward and reverse scans, leading to a hysteresis loop whose area encoded ionic memory. Figure S5 and Part III in Supporting Information further provided supporting simulations and additional discussion.

Figure 3D compares hysteretic behavior at a fixed nanochannel length (*l* = 50 μm) while varying the bridge width (i.e., the number of nanochannels, *w* = 50, 75, and 100 μm, respectively). As the number of nanochannels increased (*n* ∝ *w*), the normalized hysteresis loop area *S*_*n*_ (*S*_*n*_ = *S*_loop_/(*V*_max_·*I*_max_), *S*_loop_ $=\int_{0}^{{+V}_{\max}}IdV$ ) increased, reflecting enhanced junctional ionic accumulation/depletion enabled by multiple parallel transport pathways. However, beyond an intermediate width ( >75 μm), further increases in channel density led to a diminished enhancement of hysteresis. This saturation behavior is attributed to a reduction in the local electric field strength at individual nanochannel entrances, as the applied field is distributed across an increasing number of pathways. Such field attenuation is consistent with the field-focusing effect,^32^ where excessive channel density weakens selective ionic transport at each junction and limits further growth of hysteresis. Figure 3E examines the effect of nanochannel length at a fixed nanochannel number (approximately *n* ≈ 68 for *w* = 75 μm). Short nanochannels (*l* = 50 μm) exhibited the largest *S*_*n*_, whereas intermediate and long nanochannels (*l* = 75 and 100 μm) produced progressively smaller *S*_*n*_. Short channels facilitate rapid ionic migration and strong accumulation-depletion responses under applied bias, resulting in pronounced but relatively transient hysteretic behavior. In contrast, intermediate and long nanochannels hinder ionic transport and relaxation,^6,33^ stabilizing ionic distributions but reducing the overall magnitude of the hysteresis response. These results indicate that channel length governs the trade-off between response intensity and memory retention in nanofluidic memristors. Short nanochannels favor high response efficiency, characterized by strong and rapid hysteretic responses, whereas longer nanochannels promote transport-mediated stabilization of ionic distributions, enhancing memory retention at the expense of response magnitude.

Figure 3F summarizes the trends across nine geometrical configurations. Increasing the number of nanochannels enhances *S*_*n*_, while excessively high channel densities lead to saturation due to enhanced field focusing effect. Across all widths, shorter nanochannels consistently produce larger *S*_*n*_. Additional hysteresis characterizations for various geometries are provided in Figure S6. The effects of electrolyte concentration and scan rate on hysteretic responses were further investigated. Increasing the electrolyte concentration above 10 μM reduces the Debye length, weakening surface-charge-governed selectivity and lowering the hysteresis magnitude. Conversely, decreasing the LSV scan rate from 1 V/s to 0.025 V/s enlarged the hysteresis loop as ions had more time to redistribute (Figure S7). Collectively, these results demonstrated that memristive-like hysteresis in wrinkle-based nanochannels mainly arises from reversible, geometry-dependent ionic accumulation and relaxation rather than irreversible structural changes. Pronounced memristive behavior was achieved when short nanochannels and a sufficiently high, but not excessive, number of parallel pathways synergistically promote ICP, EDL overlap, and localized field focusing effect.

### Emulation of Synaptic Plasticity Using Wrinkle-Based Nanofluidic Memristors

Figure 4 investigates the geometry-dependent ionic transport behavior of the WNAD under pulsed electrical stimulation and its capability to emulate key features of synaptic plasticity through reversible ionic redistribution. As illustrated in Figure 4A, wrinkle-based nanochannels act as confined ionic transport pathways bridging two main microchannels. Under an applied electric field, ions traverse these nanoscale gaps, providing an analogy to neurotransmitter-mediated signal transmission across a biological synaptic cleft. Importantly, this analogy is functional rather than structural, as the observed behavior arises from controllable ionic accumulation and relaxation within confined geometries. Figure 4B,C quantify STP using PPF and PTP metrics under write pulses (3 V, 100 ms duration, 100 ms interval). PPF and PTP were defined as $\left(\frac{I_{2}-I_{1}}{I_{2}}\right)\times 100$ , and $\left(\frac{I_{10}-I_{1}}{I_{2}}\right)\times 100$ %, where *I*_1_, *I*_2_ and *I*_10_ are currents induced by first, second, 10th voltage pulses, respectively. Both metrics exhibited dependence on nanochannel geometry, consistent with the trends observed in Figure 3. In particular, an intermediate bridge width (*w* = 75 μm) combined with a short nanochannel length (*l* = 50 μm) produced the largest and most reproducible responses, indicating a trade-off between ionic accumulation capacity and transport confinement. Figure 4D,E further demonstrate potentiation-depression cycling for this geometry. Under alternating write pulses of ±3 V and read pulses of 1 V, the device exhibited stable and largely symmetric conductance modulation over repeated cycles. The conductance changes were reproducible without abrupt collapse or drift, confirming that the modulation originates from repeatable ionic redistribution rather than irreversible structural modification. Figure S8 further confirms that similar conductance modulation was consistently observed across all geometries, with channel design primarily affecting the magnitude of modulation and its persistence. While ICP is expected to be reversible over the characteristic diffusion time scale, the detailed physical origin of the observed conductance dynamics during repeated read operations remains not fully understood and requires further investigation. Figure 4F presents repeated write-read sequences applied to the WNAD (*w* = 75 μm and *l* = 50 μm). Each write sequence increased the conductance, while subsequent read pulses induced partial relaxation. Notably, the conductance did not return to its initial baseline after each cycle, resulting in a gradual upward shift of the conductance level over successive cycles. This cumulative reinforcement arises from residual ionic redistribution caused by incomplete ionic relaxation under repeated stimulation rather than a discrete transition to a permanently stored state. Instead, it reflects incremental accumulation of residual ionic redistribution enabled by incomplete relaxation between stimulation events. Such cumulative modulation demonstrates gradual analog conductance tuning under repeated programming, which is relevant for weight update operations in iontronic crossbar architectures. Figure 4G compares the effect of nanochannel geometry on cumulative conductance reinforcement. After a single stimulation cycle, all devices exhibited rapid relaxation behavior characteristic of short-lived ionic accumulation. After five consecutive cycles, however, only the device with specific WNAD maintained a substantially elevated conductance level.^11,12^ Devices with other geometries exhibited more complete relaxation, indicating that the conversion of transient ionic accumulation into a more persistent state critically depends on the cooperative balance between nanochannel number and length (Figure S9). This geometry-specific behavior is consistent with the hysteresis characteristics identified in Figures 3 and 4, underscoring the role of structural confinement in prolonging ionic memory retention through transport-mediated stabilization of ionic distributions. Figure 4H,I demonstrate that nanochannel number and length primarily govern the decay rate and retention of ionic memory under repeated read operations, rather than the initial strength of potentiation. Figure 4H examines the effect of nanochannel number while fixing the nanochannel length at *l* = 50 μm. A stimulation protocol consisting of 20 write pulses (3 V, 100 ms duration, 100 ms interval) followed by 50 read pulses (1 V, 100 ms duration, 100 ms interval) was applied. The normalized current value was defined as (*I*_*i*_ – *I*_1_)/*I*_1_, where *I*_*i*_ is the current measured at the *i*-th pulse. After completion of the read sequence, the normalized current value exhibited a nonmonotonic dependence on bridge width, with the largest retained conductance observed at *w* = 75 μm, followed by *w* = 100 μm and *w* = 50 μm. This result indicates that the decay rate of ionic memory during repeated readout is not determined solely by the number of parallel nanochannels. While wider bridge enhances ionic accumulation during the write phase, excessive channel density promotes lateral redistribution and partial relaxation of ions during read pulses, accelerating conductance decay. An intermediate width therefore minimizes relaxation-driven loss while maintaining sufficient accumulation, leading to the slowest decay and highest retained conductance. Figure 4I further elucidates this decay behavior by varying the nanochannel length while fixing the bridge width at *w* = 75 μm (approximately 68 nanochannels). Under the same write-read protocol, the normalized current remaining after the read sequence was highest for *l* = 100 μm, followed by *l* = 50 μm and *l* = 75 μm. This ordering highlights the competition between rapid ionic response and transport-mediated stabilization, shorter nanochannels facilitate efficient accumulation but allow faster relaxation, whereas longer nanochannel suppresses diffusion and slows the decay of ionic memory.

**Figure 4:**
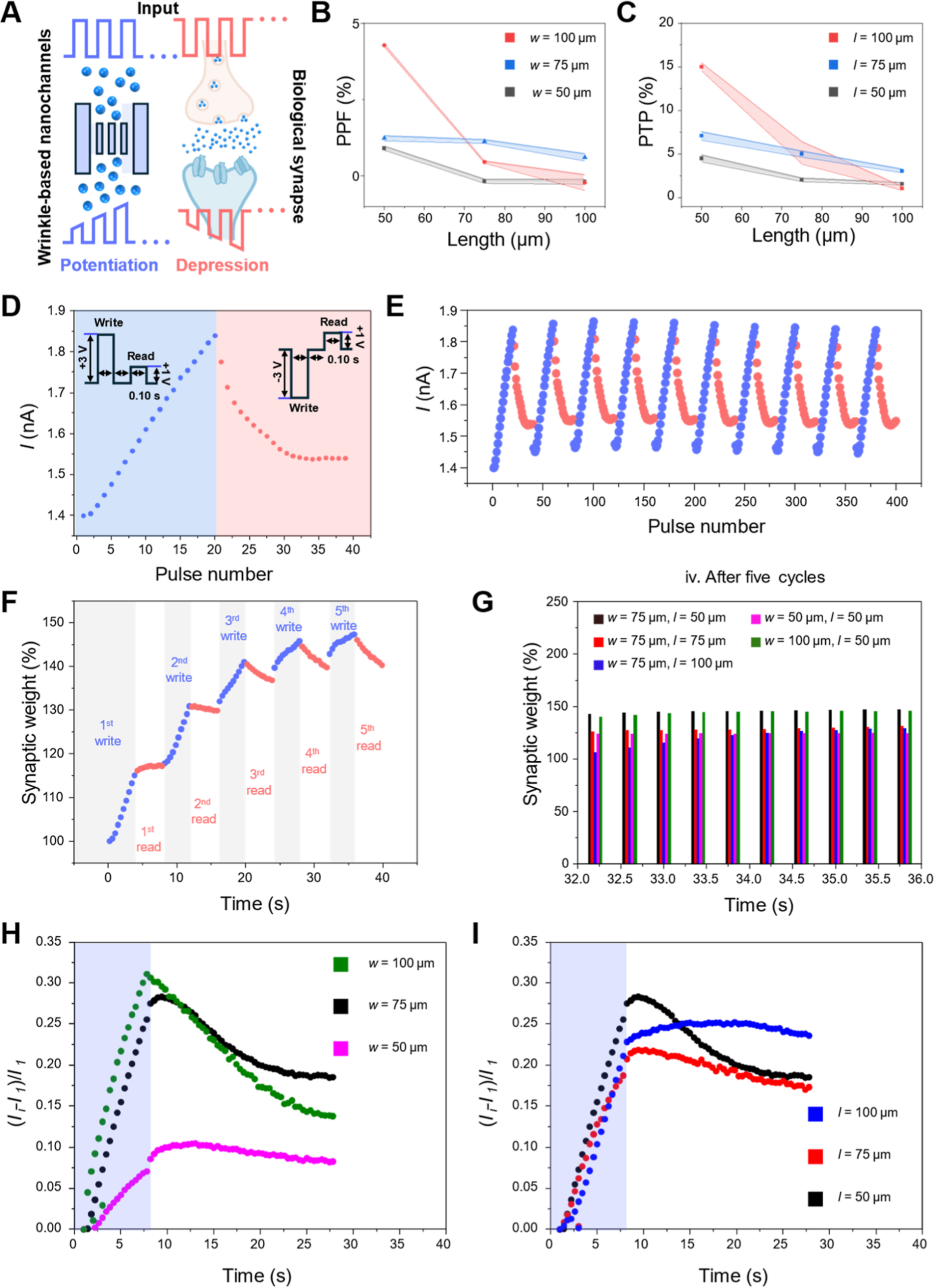
Geometry-dependent ionic plasticity and conductance modulation in WNADs. (A) Schematic illustration of wrinkle-based nanochannels acting as confined ionic transport pathways, serving as artificial synaptic elements under pulsed electrical stimulation. (B,C) Quantification of short-term plasticity (STP) using paired-pulse facilitation (PPF) and post-tetanic potentiation (PTP) under write pulses (3 V, 100 ms duration, 100 ms interval). (D,E) Reproducible potentiation-depression cycling under alternating write pulses (±3 V) and read pulses (1 V). (F) Representative conductance evolution during repeated write-read sequences for the WNAD. (G) Comparison of cumulative conductance reinforcement for devices with different nanochannel geometries after repeated stimulation cycles. (H,I) Normalized current response for devices with different bridge widths (*w* = 50, 75, 100 μm) at fixed nanochannel length (*l* = 50 μm), and different nanochannel lengths (*l* = 50, 75, 100 μm) at a fixed bridge width (*w* = 75 μm, ∼68 nanochannels), measured after a stimulation protocol consisting of 20 write pulses (3 V, 100 ms duration, 100 ms interval) followed by 50 read pulses (1 V, 100 ms duration, 100 ms interval).

### Geometry-Dependent Ionic Potentiation and Retention

Figure 5A shows the temporal evolution of conductance after applying a single write pulse (3 V, 10 s duration, 100 ms interval), followed by read pulses (1 V, 100 ms duration, 1.9 s interval) used only to monitor the conductance state. After the write pulse induces ionic accumulation within the wrinkle-based nanochannels, the conductance gradually relaxes toward the baseline over time. The decay profiles clearly depend on nanochannel geometry, indicating that memory retention is governed by structural confinement. Additional decay characterizations for various geometries are provided in Figure S10. Figure 5B summarizes the decay times as a function of nanochannel geometry. For devices with a fixed length of *l* = 100 μm, the decay time increased monotonically with bridge width, from 60.7 s (*w* = 50 μm) to 64.1 s (w = 75 μm), and 79.4 s (*w* = 50 μm). Notably, this increase does not indicate a suppression of relaxation dynamics, rather, wider bridges enable stronger write-induced ionic accumulation and a larger initial conductance increase, resulting in a longer time required for the conductance to relax back to the baseline. When varying the length at a fixed width of *w* = 75 μm, the decay time increased markedly from 26.1 s (*l* = 50 μm) to 43.4 s (*l* = 75 μm) and 64.1 s (*l* = 100 μm). In this case, the length dependence reflects diffusion-limited relaxation, where longer nanochannels intrinsically slow ionic diffusion and extend the characteristic retention time (τ) scales as τ ∼ *L* ^2^/*D*, where *D* denotes the ionic diffusivity within the confined nanochannels. Taken together, these results clarify that nanochannel width and length influence retention through distinct physiochemical mechanisms. Increasing bridge width primarily enhances the magnitude of write-induced potentiation, thereby elevating the initial conductance state, whereas increasing nanochannel length directly slows relaxation by limiting diffusive ionic transport. This separation of accumulation-dominated and relaxation-dominated effects underscores the deterministic tunability of ionic memory through structural design.

**Figure 5:**
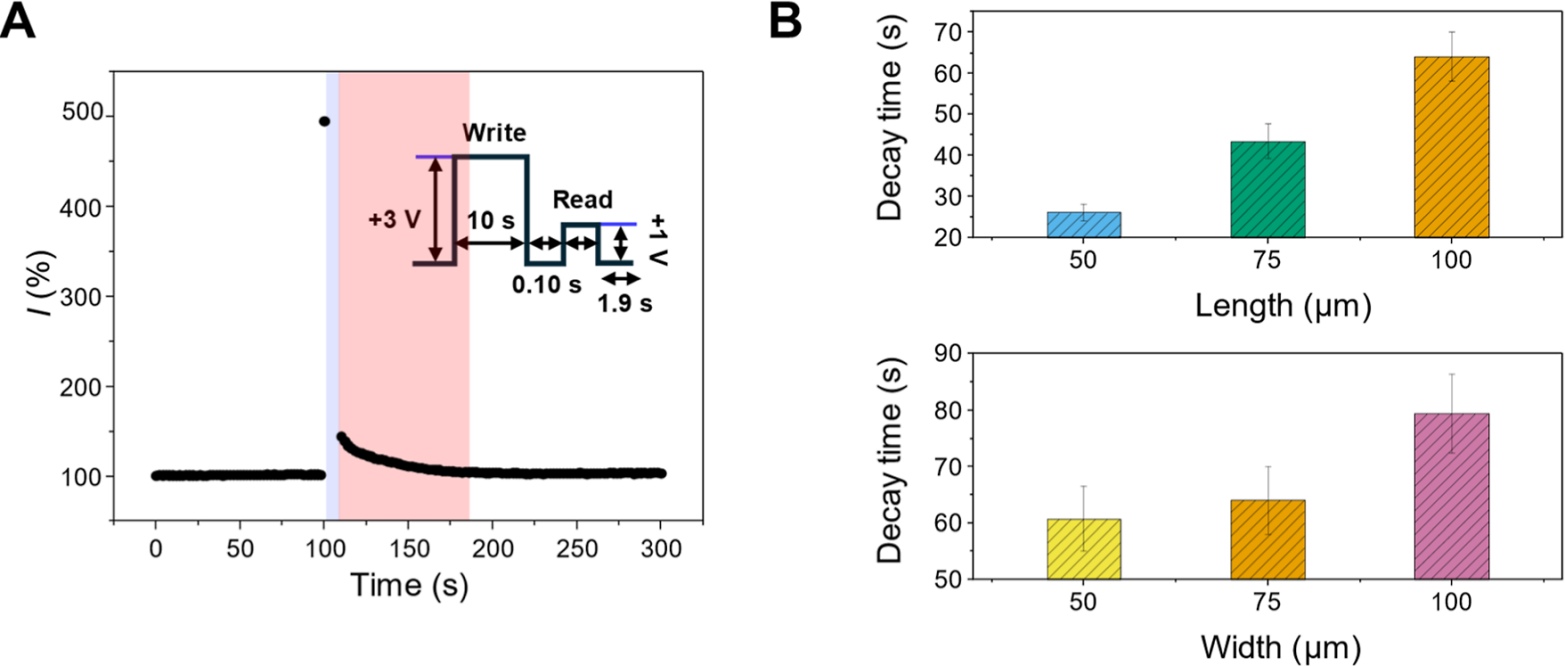
Geometry-dependent memory decay characteristics of WNAD. (A) Temporal evolution of conductance following a single write pulse (3 V, 10 s duration, 100 ms interval), monitored using intermittent read pulses (1 V, 100 ms duration, 1.9 s interval). (B) Memory decay times as a function of bridge width and length.

## Discussion

This study demonstrated that wrinkle-based nanochannels enabled geometry-regulated ionic memory through reversible ion accumulation and relaxation. By tuning nanochannel number and length, the WNAD exhibited controllable transitions between transient and more persistent ionic states, confirming the feasibility of geometry-driven ionic memory. The current WNAD architecture relied on stochastic alignment between the bridge channel and the wrinkled substrate during bonding, which introduced local variations in wrinkle morphology and effective nanochannel number. Despite this variability, optimized fabrication and bonding procedures yielded consistent memristive and synaptic-like behaviors across multiple devices, indicating robustness of the underlying ionic mechanisms. Nevertheless, spatial heterogeneity inherent to wrinkle formation may have influenced local electric field distributions and transport pathways, contributing to variations in relaxation behavior. However, the relatively short long-term memory (LTM)-like decay times observed in Figure 5 are not solely attributable to wrinkle nonuniformity. Instead, they are fundamentally constrained by the structural scale of nanochannels and the transport properties of the electrolyte. In the WNAD, the effective channel dimensions are determined by the wrinkle wavelength and photolithographically defined bridge geometry, which limit the degree of nanoscale confinement and allow diffusion-mediated ionic relaxation on relatively short time scales. In addition, the ionic concentration and conductivity of the electrolyte govern ionic diffusivity and redistribution rates, such that higher ionic mobility accelerates relaxation even within geometrically confined channels. Consequently, the observed retention behavior reflects a coupled effect of channel dimensions and electrolyte transport properties, rather than geometric confinement alone. Compared with other reported nanofluidic memristive systems that achieve extended retention through sub-nm confinement, chemically gated nanopores, or low-mobility ionic media, the present platform prioritizes geometric programmability and fabrication simplicity. Guided wrinkle alignment or strain-programmed wrinkling, combined with higher resolution patterning and electrolyte or surface-charge engineering, could therefore improve uniformity, strengthen confinement, and bridge the retention gap while preserving the structural tunability demonstrated in this work. Beyond geometric control, introducing dynamically tunable surface charge interfaces,^34^ for example, via polyelectrolyte multilayers,^14^ pH-responsive coatings,^35^ ion-selective hydrogels,^13^ or redox-active functional groups,^36,37^ could have expanded device functionality by enabling adaptive modulation of ionic conductance. Such approaches may have supported more complex history-dependent behaviors without relying solely on static structural parameters. Although the nanochannel dimensions exceeded those of biological synapses, the WNAD reproduced key functional features such as reproducible potentiation-depression cycles, cumulative reinforcement under repeated stimulation, and geometry-dependent stabilization of ionic states. Differences in transport regimes and time scales relative to biological systems remained, indicating that shorter conduction pathways and chemically or electrically gated transport could further enhance biomimetic operation. Overall, the WNAD established a proof-of-concept for geometry-regulated ionic memory. Further progress in wrinkle alignment, nanoscale precision, surface charge programmability, mechanistic modeling, and network-level integration will be critical for advancing toward scalable and reliable iontronic systems for brain-inspired information processing.^11,12,38^

## Conclusions

In summary, we demonstrated wrinkle-based nanochannels as a versatile and geometrically tunable platform, referred to as the wrinkle-based nanochannel array device, for geometry-dependent ionic memory and synaptic functions. By integrating wrinkle structures within a hybrid PDMS/OSTEMER microfluidic chip, nanochannels were selectively preserved at the OSTEMER–OSTEMER interface, while collapse at the PDMS-OSTEMER interface spatially confined ionic transport to the designed bridge region. Systematic characterization revealed that the memristive hysteresis behavior depended strongly on geometric parameters, particularly the width and length of the bridge channel, which directly determine the number and length of the nanochannels. The configuration (*w* = 75 μm, *l* = 50 μm) exhibited the largest hysteresis loop area, attributed to enhanced accumulation-depletion dynamics and field-focusing effects. The retention behavior was further shown to depend on nanochannel length, following diffusion-limited scaling, which highlights the direct coupling between device geometry and ionic relaxation dynamics. Beyond ICP and hysteretic transport, the wrinkle-based nanochannels exhibited a range of synaptic responses under pulsed electrical stimulation, including reversible potentiation-depression cycling, PPF, PTP, and geometry-dependent cumulative reinforcement over repeated stimulation cycles. Importantly, these behaviors originated from reversible ionic redistribution and diffusion-limited relaxation. While repeated stimulation led to progressively more persistent conductance states, the observed retention remained within a long-lived, metastable regime rather than nonvolatile LTM. Overall, these results establish wrinkle-based nanochannels as a structurally programmable iontronic platform in which ionic transport characteristics, hysteresis strength, and memory time scales can be deterministically tuned through geometry alone. Coupled with the scalability and low-cost nature of wrinkle-based nanofabrication, the WNAD provides a practical foundation for advancing nanofluidic iontronics and for developing artificial synaptic elements and neuromorphic architectures based on controllable ionic dynamics, while clearly delineating the physical limits of diffusion-governed ionic memory.

## Materials and Methods

### Materials and Reagents

Negative photoresists, SU-8 2010 and 2025 (MicroChem, Westborough, MA, USA), polydimethylsiloxane (PDMS, Sylgard 184, Dow Corning, Midland, MI, USA), and an off-stoichiometry thiol–ene polymer resin (OSTEMER 322 Crystal Clear, Mercene Laboratories AB, Stockholm, Sweden) were used for the fabrication of WNADs. To reduce surface energy and facilitate mold release, the silicon mold was treated with trichloro­(1*H*,1*H*,2*H*,2*H*-perfluorooctyl)­silane (Merck, Darmstadt, Germany). Sulforhodamine-B sodium salt (Merck, Darmstadt, Germany) was used to characterize wrinkle-based nanochannels, while a 10 μM potassium chloride (KCl) solution containing 10 μM fluorescein isothiocyanate (FITC) was used to visualize ICP by tracking fluorescence-based ion accumulation and depletion. KCl (Merck, Darmstadt, Germany) served as the electrolyte, and platinum electrodes (BASMW1032, Merck, Darmstadt, Germany) were used for electrical measurements. Unless otherwise specified, the electrolyte concentration was fixed at 10 μM KCl. Wrinkles were transferred onto 250 μm-thick polyethylene terephthalate (PET) films using NOA63 adhesive (Norland Products, Jamesburg, NJ, USA) and a cylindrical glass bending mold with an 84 mm diameter. Unless otherwise noted, chemicals were purchased from Merck.

### Device Fabrication

Microfluidic molds were fabricated by photolithography (MA-6, Suss Microtec, Munich, Germany) using a two-level SU-8 process on silicon wafers. PDMS replicas were prepared by casting and curing Sylgard 184 prepolymer (10:1 base to curing agent ratio) at 70 °C for 2 h. To enhance surface wettability before bonding, O_2_ plasma treatment (Cute-MP, Femto Science, Gyeonggi, Korea) was performed. In the PDMS/Poly­(vinyl alcohol) (PVA) bilayer system, the PVA thickness was controlled by adjusting the spin-coating speed (SPIN-1200D, Midas System, Daejeon, Korea). Contact angles were measured using a goniometer (Smart Drops SDL200TEZD, FemtoFAB, Gyeonggi, Korea) to confirm surface energy modification. Two UV curing systems were employed under distinct exposure conditions depending on the target structure. OSTEMER resin injected into the hybrid OSTEMER/PDMS chip was cured using a custom-built UV system (ANUP5252L, Panasonic Industry, Tokyo, Japan) at a power of approximately 300 mW for 20 s to ensure complete polymerization and facilitate demolding. Wrinkle patterns replicated in OSTEMER were cured using a secondary UV lamp (LF-215L, Novolab, Geraardsbergen, Belgium) for 5 min at about 3 mW to preserve nanoscale wrinkle fidelity. UV intensity was calibrated using a power meter (PM100USB, Thorlabs, Newton, NJ, USA).

### Characterization and Electrical Measurements

Wrinkle morphology and periodicity were characterized using a scanning electron microscope (SEM, S-4800, Hitachi, Tokyo, Japan) and atomic force microscopy (AFM, D3100, Veeco, Plainview, NY, USA). Fluorescence images were acquired using an inverted fluorescence microscope (Eclipse Ti–U, Nikon, Tokyo, Japan) equipped with a charge-coupled device (CCD) camera (ORCA R2, Hamamatsu Photonics, Shizuoka, Japan). Electrical characterization was performed using a SourceMeter (2600B, Keithley, Solon, OH, USA). Current–voltage (*I*–*V*) characteristics were recorded at a typical scan rate of 50 mV/s, and linear sweep voltammetry (LSV) measurements were controlled through a custom Python script that also computed normalized hysteresis loop areas. Voltage pulse protocols were applied via custom LABVIEW software for evaluating potentiation-depression and synaptic plasticity behavior. Fluorescence imaging was conducted to observe ion concentration and depletion behavior under applied bias, using a 10 μM FITC–KCl mixture. ImageJ (National Institutes of Health, Bethesda, MD, USA) was employed for fluorescence intensity quantification, and OriginPro 2020 (OriginLab Corp., Northampton, MA, USA) was used for data plotting and statistical analysis.

### Numerical Simulations

Numerical simulations were performed using COMSOL Multiphysics (version 5.3, COMSOL Inc., Stockholm, Sweden) to analyze coupled electro-diffusion phenomena. The Poisson-Nernst–Planck (PNP) equations were solved under time-dependent conditions to model ion transport, electric potential distribution within the wrinkle-based nanochannels. Boundary conditions included surface charge density, electrolyte concentration, and applied bias corresponding to experimental parameters. The simulation outputs were used to correlate experimentally observed hysteresis behaviors with ionic accumulation-depletion dynamics across different nanochannel geometries.

## Supplementary Material



## Data Availability

All the data required to evaluate the results of the study are presented in the paper and/or as Supporting Information. Additional data related to this study can be obtained from the authors upon reasonable request.

## Notes

Wrinkle fabrication and buckling mechanism (Figure S1); surface wettability and selective OSTEMER filling (Figure S2); fabrication of the PDMS/OSTEMER hybrid device (Figure S3); fluorescence visualization of ion concentration polarization (Figure S4); numerical simulations of ion transport and hysteresis (Figure S5); geometry-dependent I–V hysteresis characteristics (Figure S6); effects of electrolyte concentration and scan rate (Figure S7); potentiation-depression cycling behavior (Figure S8); conductance evolution characteristics (Figure S9); geometry-dependent conductance decay (Figure S10); and Supporting Information notes (PDF)

## References

[1] Schuman C. D. (2022). Opportunities for neuromorphic
computing algorithms and applications. Nat.
Comput. Sci..

[2] Dai S., Liu X., Liu Y., Xu Y., Zhang J., Wu Y., Cheng P., Xiong L., Huang J. (2023). Emerging Iontronic
Neural Devices for Neuromorphic Sensory Computing. Adv. Mater..

[3] Mei T. (2025). Ion–Electron
Interactions in 2D Nanomaterials-Based Artificial
Synapses for Neuromorphic Applications. ACS
Nano.

[4] Noh Y., Smolyanitsky A. (2024). Synaptic-like
plasticity in 2D nanofluidic memristor
from competitive bicationic transport. Sci.
Adv..

[5] Yao P. (2020). Fully
hardware-implemented memristor convolutional neural network. Nat..

[6] Kamsma T. M., Kim J., Kim K., Boon W. Q., Spitoni C., Park J., van Roij R. (2024). Brain-inspired
computing with fluidic iontronic nanochannels. Proc. Natl. Acad. Sci. U. S. A..

[7] Li Z., Feng J., Xiao J., Leuski A., Noy A. (2026). Synaptic Functionality
and Neuromorphic Information Processing in Membrane Ion Channel Junctions. Adv. Mater..

[8] Li Z., Myers S. K., Xiao J., Li Y., Noy N., Leuski A., Noy A. (2025). Neuromorphic ionic computing in droplet
interface synapses. Sci. Adv..

[9] Zhang M. (2025). Nanofluidic Volatile Threshold Switching Ionic Memristor:
A Perspective. ACS Nano.

[10] Xu G. (2025). Angstrom-Scale-Channel Iontronic Memristors for Neuromorphic
Computing. ACS Appl. Mater. Interfaces.

[11] Wang J., Jiang Y., Xiong T., Lu J., He X., Yu P., Mao L. (2025). Optically Modulated
Nanofluidic Ionic Transistor for
Neuromorphic Functions. Angew. Chem. Int. Ed..

[12] Song R. (2025). Nanofluidic Memristive
Transition and Synaptic Emulation in Atomically
Thin Pores. Nano Lett..

[13] Zhang Z. (2024). Geometrically Scalable
Iontronic Memristors: Employing Bipolar Polyelectrolyte
Gels for Neuromorphic Systems. ACS Nano.

[14] Li J., Li M., Zhang K., Hu L., Li D. (2023). High-Performance Integrated
Iontronic Circuits Based on Single Nano/Microchannels. Small.

[15] Bu Y., Ahmed Z., Yobas L. (2019). A nanofluidic memristor based on
ion concentration polarization. Analyst.

[16] Law C. S., Wang J., Nielsch K., Abell A. D., Bisquert J., Santos A. (2025). Recent advances in
fluidic neuromorphic computing. Appl. Phys.
Rev..

[17] Yu L. (2024). Bioinspired nanofluidic
iontronics for brain-like computing. Nano Res..

[18] Chung S. (2008). Non-Lithographic Wrinkle
Nanochannels for Protein Preconcentration. Adv.
Mater..

[19] Malachias A. (2008). Wrinkled-up nanochannel networks: long-range ordering, scalability,
and X-ray investigation. ACS Nano.

[20] Lee J. H. (2020). Direct nanofluidic channels
via hardening and wrinkling of thin polymer
films. Nanoscale.

[21] Park S.-m. (2009). A method for nanofluidic device prototyping using elastomeric collapse. Proc. Natl. Acad. Sci. U. S. A..

[22] Huh D. (2007). Tuneable elastomeric nanochannels for nanofluidic manipulation. Nat. Mater..

[23] Xiong T. (2023). Neuromorphic functions with a polyelectrolyte-confined fluidic memristor. Sci..

[24] Bae J., Wu R., Kim T. (2023). Fabricating
and Laminating Films with Through-Holes
and Engraved/Protruding Structures for 3D Micro/Nanofluidic Platforms. Small Methods.

[25] Bae J., Jeon H., Kim T. (2024). Full-Combinatorial Concentration
Gradient Array with 3D Micro/Nanofluidics for Antibiotic Susceptibility
Testing. Anal. Chem..

[26] Cho H. (2007). How the capillary burst
microvalve works. J.
Colloid Interface Sci..

[27] Choi E. (2016). High Current Ionic Diode Using Homogeneously Charged
Asymmetric Nanochannel
Network Membrane. Nano Lett..

[28] Seo D., Park G., Kim J., Kim T., Park J. (2025). Enhancement
of nanofluidic ionic current via plasmon-mediated effect. Sens. Actuators, B.

[29] Kim J., Cho I., Lee H., Kim S. J. (2017). Ion Concentration Polarization by
Bifurcated Current Path. Sci. Rep..

[30] Aubrecht P. (2024). Performance and biocompatibility of OSTEMER
322 in cell-based microfluidic
applications. RSC Adv..

[31] Ismail A., Nam G. H., Lokhandwala A., Pandey S. V., Saurav K. V., You Y., Jyothilal H., Goutham S., Sajja R., Keerthi A. (2025). Programmable memristors with two-dimensional nanofluidic channels. Nat. Commun..

[32] Sebastian J., Green Y. (2023). Electrical Circuit
Modeling of Nanofluidic Systems. Adv. Phys.
Res..

[33] Zhang Y., Liu L., Qiao Y., Yao T., Zhao X., Yan Y. (2025). Confinement
of ions within graphene oxide membranes enables neuromorphic artificial
gustation. Proc. Natl. Acad. Sci. U. S. A..

[34] Emmerich T. (2024). Nanofluidic logic with
mechano-ionic memristive switches. Nat. Electron..

[35] Paulo G., Sun K., Di Muccio G., Gubbiotti A., Morozzo della Rocca B., Geng J., Maglia G., Chinappi M., Giacomello A. (2023). Hydrophobically
gated memristive nanopores for neuromorphic applications. Nat. Commun..

[36] Xie B., Xiong T., Guo G., Pan C., Ma W., Yu P. (2025). Bioinspired ion-shuttling memristor
with both neuromorphic functions
and ion selectivity. Proc. Natl. Acad. Sci.
U. S. A..

[37] Zhang B., Fan F., Xue W., Liu G., Fu Y., Zhuang X., Xu X. H., Gu J., Li R. W., Chen Y. (2019). Redox gated
polymer memristive processing memory unit. Nat.
Commun..

[38] Abraham W. C., Bear M. F. (1996). Metaplasticity:
the plasticity of synaptic plasticity. Trends
Neurosci..

