# Supplementary material for: Wrinkle-Assisted Nanofluidic Memristors for Geometry-Dependent Ionic Memory
Source: ACS Nano. 2026 Apr 3;20(14):10997–1007. doi: 10.1021/acsnano.5c20258 (PMC13085843; doi:10.1021/acsnano.5c20258)
Supplement: Supplementary file 1 [file nn5c20258_si_001.pdf]

Supplementary Information

Wrinkle-Assisted Nanofluidic Memristors for Geometry-dependent Ionic Memory

*Minsu Kwon[1], Dongwoo Seo[1], and Taesung Kim[1,2] **

[1]Department of Mechanical Engineering, Ulsan National Institute of Science and Technology (UNIST), 50 UNIST-Gil, Ulsan 44919, Republic of Korea.

[2]Department of Biomedical Engineering, Ulsan National Institute of Science and Technology (UNIST), 50 UNIST-Gil, Ulsan 44919, Republic of Korea.

***Correspondence**

Taesung Kim

Department of Mechanical Engineering

Ulsan National Institute of Science and Technology (UNIST)

50 UNIST-gil, Ulsan 44919, Republic of Korea

E-mail: tskim@unist.ac.kr

Tel: +82-52-217-2313

Fax: +82-52-217-2409

Table of Contents

**Supplementary Notes**	Buckling theory to generate wrinkle surfaces	S-3
	Ion concentration polarization (ICP) through wrinkle-based nanochannels	S-4
	Numerical simulations of ICP in the WNAD	S-5
**Figure S1**	Fabrication and mechanism of wrinkle surface formation	S-9
**Figure S2**	Wettability of various substrates and selective filling of OSTEMER resin into the bridge channel in the WNAD	S-10
**Figure S3**	Fabrication process of a PDMS/OSTEMER hybrid device	S-12
**Figure S4**	Time-lapse fluorescence imaging of ICP in wrinkle-based nanochannels	S-13
**Figure S5**	Numerical simulation results of ion transport and hysteresis behavior	S-14
**Figure S6**	Hysteresis loops in I-V curves of the WNAD under different bridge channel geometrical dimensions	S-15
**Figure S7**	Effect of electrolyte concentration and scan rate on the hysteresis loop area	S-16
**Figure S8**	Potentiation-depression cycling of the WNAD under different geometrical dimensions	S-17
**Figure S9**	Conductance evolution of the WNAD with different geometrical dimensions	S-18
**Figure S10**	Geometry-dependent decay characteristics	S-19

Supplementary Notes

Part I. Buckling theory to generate wrinkle surfaces

Surface wrinkles form when a thin, stiff film bonded to a soft substrate experiences compressive stress exceeding a critical threshold. Once this stress surpasses the elastic stability limit, the initially flat bilayer surface relieves its stored strain energy through periodic buckling, producing ordered wrinkle patterns that can serve as nanoscale templates for ionic nanochannels. The overall fabrication sequence is illustrated in Figure S1A. A PDMS/PVA bilayer was first mounted on a bending mold coated with a thin NOA63 layer on a PET substrate (Figure S1A-i). When the mold curvature was applied, the outer PVA film underwent compressive strain due to modulus mismatch between the stiff PVA layer and the soft PDMS substrate, thereby forming spontaneous wrinkle patterns (Figure S1A-ii). These wrinkles were subsequently transferred onto the NOA63-coated PET, and after demolding, a replicated wrinkle pattern supported on PET was obtained (Figure S1A-iii, iv). PDMS was then cast against the wrinkled NOA63 surface to create a PDMS master mold containing the inverse wrinkle pattern (Figure S1A-v). Using this PDMS master mold, the wrinkle features were replicated onto an OSTEMER layer by UV nanoimprinting and cured under UV to solidify the final structure (Figure S1A-vi). After demolding, the replicated wrinkle structure on OSTEMER, supported by a glass substrate, was obtained (Figure S1A-vii, viii). This multilayer transfer process ensures high-fidelity replication of wrinkle morphology from the original PDMS/PVA bilayer to the final OSTEMER substrate while preserving nanoscale amplitude and periodicity required for iontronic and nanofluidic applications. The fundamental mechanics of wrinkle generation are schematically shown in Figure S1B. When the bilayer system is subjected to bending, the outer surface undergoes compression, while the inner surface experiences tension. Once the compressive strain exceeds a critical strain (e_c), periodic surface buckling occurs to minimize the total elastic energy. The wrinkle geometry, defined by the

wavelength (λ) and amplitude (h), can be predicted using classical wrinkling theory. Following the relation $\lambda = 2\pi t_f \sqrt[3]{E_f/3E_s}$, the λ depends on the film thickness (t_f, e.g., PVA) and the elastic modulus contrast between the film (E_f) and the substrate (E_s, e.g., PDMS). This indicates that a stiffer film or a softer substrate leads to a longer wrinkle wavelength. The h depends on the applied pre-strain (e_0) relative to e_c, described as $h = t_f\sqrt{\frac{e_0-e_c}{e_c}}$. Thus, the wrinkle amplitude increases with higher applied strain, allowing tunable control of nanochannel confinement depth. The critical strain itself is defined as $e_c = \frac{1}{4}\left(\frac{3E_s}{E_f}\right)^{\frac{2}{3}}$, which reflects the threshold beyond which surface buckling becomes energetically favorable. Together, these relationships collectively provide a quantitative framework for designing wrinkle geometries by adjusting material properties and mechanical strain. Decreasing E_s or increasing E_f enlarges λ, whereas increasing e_0 amplifies h. Both parameters directly determine the effective nanochannel cross-section and ionic confinement characteristics. In this study, the theoretical predictions guided the selection of PDMS/PVA bilayer composition and pre-strain parameters, producing wrinkle nanochannels with $\lambda \approx 1.1$ μm and $h \approx 200$ nm. These nanoscale features were confirmed by AFM analysis (Figure 2E) and exhibited excellent agreement with classical wrinkling theory. The ability to tune both λ and h through material and mechanical control provides a versatile route to manipulate ionic transport behavior, electric double-layer overlap, and memristive performance in wrinkle-based nanochannel array devices (WNADs).

Part II. Ion concentration polarization (ICP) through wrinkle-based nanochannels

Under a forward bias, a distinct ion depletion zone rapidly developed near the anodic junction immediately after voltage application (Figure S4A). At $t = 2$ min, the depletion front extended further into the microchannel, and at $t = 4$ min, a sharp and well-defined depletion boundary had formed. The pronounced boundary indicated amplification of the local electric

field and the onset of electroconvection at the depletion front. On the cathodic side, an ion-accumulation region gradually appeared. Although the overall ionic strength increased, the negatively charged FITC molecules were excluded from this zone due to preferential cation accumulation. Consequently, the fluorescence profile exhibited a blurred boundary, as if FITC had been repelled from the accumulation region. When the polarity was reversed, the spatial symmetry of ICP was also reversed (Figure S4B). At t = 0 min, the fluorescence distribution remained uniform, but at t = 2 min, a depletion zone emerged near the GND side, while accumulation developed toward the opposite reservoir (i.e., GND reservoir in the figure). After t = 4 min, both the depletion and accumulation zones became clearly distinguishable, demonstrating that the formation and migration of these regions were entirely reversible with respect to the applied bias polarity. Collectively, these time-resolved fluorescence images confirmed the dynamic, bias-dependent development of depletion and accumulation within the wrinkle-based nanochannels, verifying that ion concentration polarization occurs reversibly and directionally in response to external electric fields.

Part III. Numerical simulations of ICP in the WNAD

The simulations were performed using a two-dimensional (2D) model to elucidate the ionic transport behavior and hysteresis mechanisms within the WNAD (Figure S5). The computational geometry consisted of two opposing microchannels each 25 μm wide and 25 μm long, connected by wrinkle-based nanochannels 200 nm wide and 50 μm long. A time-dependent potential was applied at the left microchannel boundary (biased side), while the right microchannel was grounded. This configuration enabled direct observation of ICP and nonlinear current response through the coupled Poisson-Nernst-Planck (PNP) equations, incorporating Gouy-Chapman (GC) boundary conditions to capture the evolution of the electric

double layer (EDL). The electrolyte concentration was set to 10 μM KCl. The Debye length was estimated using the standard relation,

$$\lambda_D = \sqrt{\frac{\varepsilon_0 \varepsilon_r RT}{2F^2 I}}$$

Where ε_0, ε_r, R, T, F, and I are the vacuum permittivity, the relative permittivity of water, the gas constant, the absolute temperature, Faraday constant, and the ionic strength respectively. The calculated Debye length was approximately 96 nm, which is comparable to the 200 nm nanochannel depth. This indicates strong EDL overlap, implying that surface conduction dominates ionic transport and drives nonlinear current-voltage (I-V) behavior. A linear sweep voltammetry (LSV) of 0 → 1 → 0 → -1 → 0 V was applied to probe dynamic hysteresis characteristics.

The electrostatic potential ϕ and ion concentrations c_i were determined by the coupled PNP equations.

$$\nabla^2 \phi = -\frac{F}{\varepsilon_0 \varepsilon_r} \sum_i z_i c_i$$

Where z_i and c_i denote the valence and concentration of ion species i (i.e., K^+ or Cl^-), respectively. The electric field is defined as $E = -\nabla\phi$. At the channel walls, the boundary condition follows:

$$-n \cdot (\varepsilon_0 \varepsilon_r \nabla \phi) = \sigma$$

where σ is the surface charge density, given by the Gouy-Chapman equation:

$$\sigma = -\sqrt{8\varepsilon_0 \varepsilon_r RTc}\, \sinh\left(\frac{F\xi}{2RT}\right)$$

Here, ξ is the zeta potential and c is the bulk electrolyte concentration. The flux of each ionic species is expressed as:

$$J_i = -D_i \nabla c_i - z_i \frac{D_i F}{RT} c_i \nabla \phi$$

where D_i is the diffusion coefficient. The time evolution of the concentration field is governed by:

$$\frac{\partial c_i}{\partial t} = -\nabla \cdot J_i$$

No flux boundary conditions $-n \cdot J_i = 0$, were applied at all solid surfaces. The reservoir boundaries were modeled with time-dependent ionic concentrations, which allowed natural formation of depletion and accumulation in response to applied bias, a condition that more accurately reproduces experimental ICP behavior that static concentration boundaries.

The simulated I-V characteristics of wrinkle-based nanochannels are shown in Figures S5A–B. For a single nanochannel, the I-V curve displayed an almost linear response with minimal nonlinearity and negligible hysteresis, indicating that polarization was spatially localized and insufficient to induce memory effects (Figure S5A). In contrast, five parallel nanochannels exhibited pronounced nonlinearity and a distinct hysteresis loop (Figure S5B), arising from enhanced ICP and interchannel coupling that broadened the depletion region and delayed charge relaxation. These results demonstrated that the number of channels critically governs ionic nonlinearity and hysteresis strength. Due to computational constraints in COMSOL Multiphysics, simulations were conducted for up to five nanochannels, and the linear sweep voltammetry (LSV) range was limited to ±1 V. Under a +1 V bias, K^+ ions accumulated inside the nanochannel, while Cl^- ions were excluded from the nanochannel, forming a strong depletion zone near the left microchannel and an accumulation zone near the right side (Figure S5C). Upon reversing the bias to -1 V, the ionic distributions inverted, accumulation occurred on the left side and depletion on the right (Figure S5D). Because the nanochannel exhibits cation-selective transport, the concentration of K^+ within the channel consistently exceeded that of Cl^- under both polarities. The observed hysteresis arises from the asymmetric EDL charging and discharging, combined with dynamic variation of surface charge

density during voltage reversal. Overall, the PNP-GC framework successfully reproduced the nonlinear ion transport and hysteretic behavior observed in wrinkle-based nanochannels. The results confirm that increasing the number of wrinkle-based nanochannels amplifies both nonlinearity and hysteresis, verifying that surface charge-regulated ion transport governs the memristive characteristics of the WNAD.

Supplementary Figures

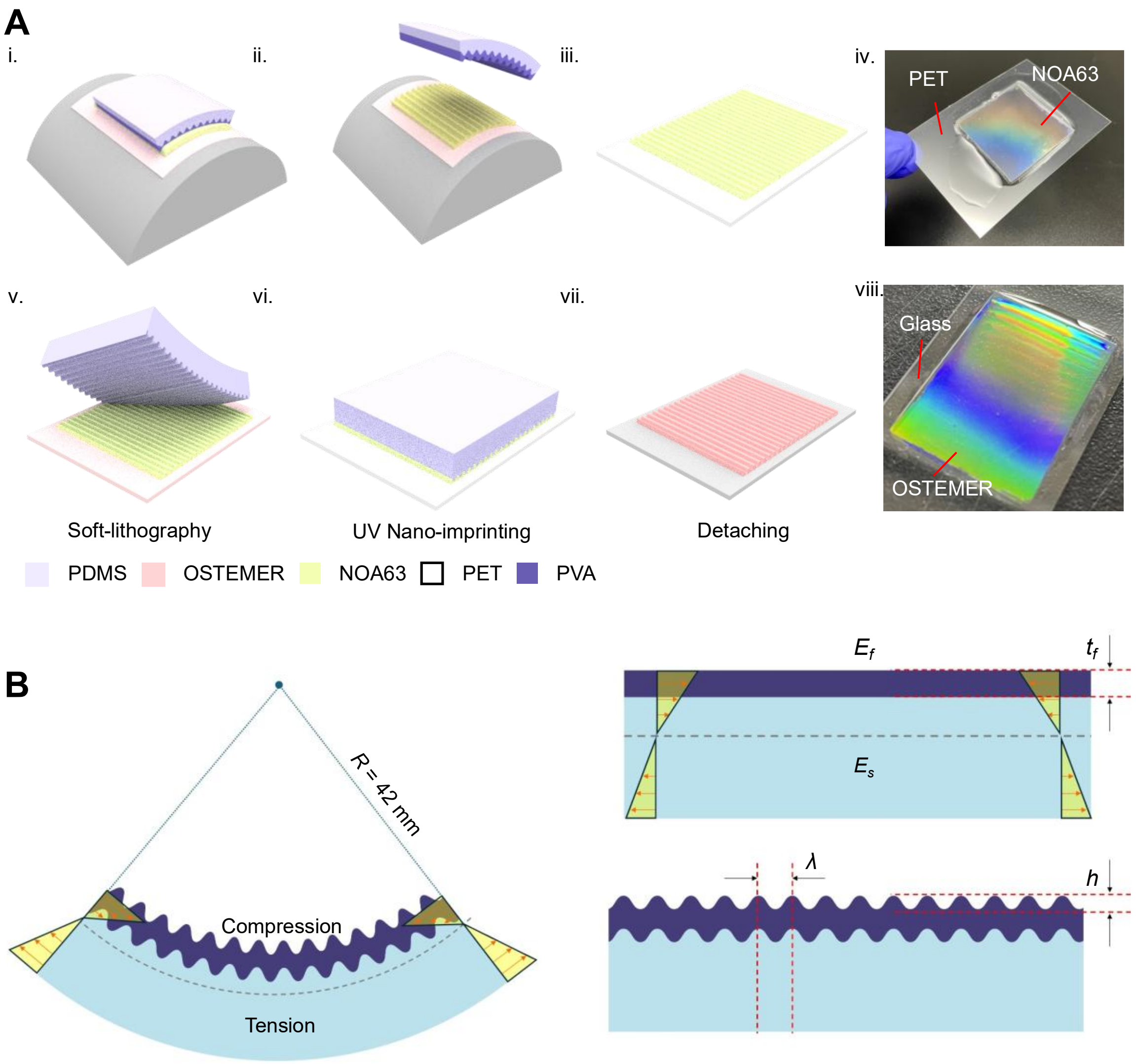

Figure S1. Fabrication and mechanism of wrinkle surface formation. (A) Fabrication of a wrinkle surface. (i) A PDMS/PVA bilayer system was placed on a bending mold with a PET substrate coated with NOA63. (ii) Wrinkles formed on the PDMS/PVA surface were transferred onto the NOA63 layer. (iii) The replicated wrinkle structure on NOA63 supported by PET was obtained after detaching from the bending mold. (iv) Photograph of the wrinkle surface on NOA63/PET. (v) PDMS was cast onto the NOA63 wrinkle surface to fabricate a PDMS master mold with replicated wrinkle features. (vi) Wrinkles formed on the PDMS master mold were transferred onto the OSTEMER layer and subsequently UV-cured (UV Nano-imprinting) to complete the replication. (vii) The replicated wrinkle structure on OSTEMER supported by glass was obtained after detaching from the PDMS mold. (viii) Photograph of the wrinkle surface on OSTEMER. (B) Mechanism of the surface wrinkle formation.

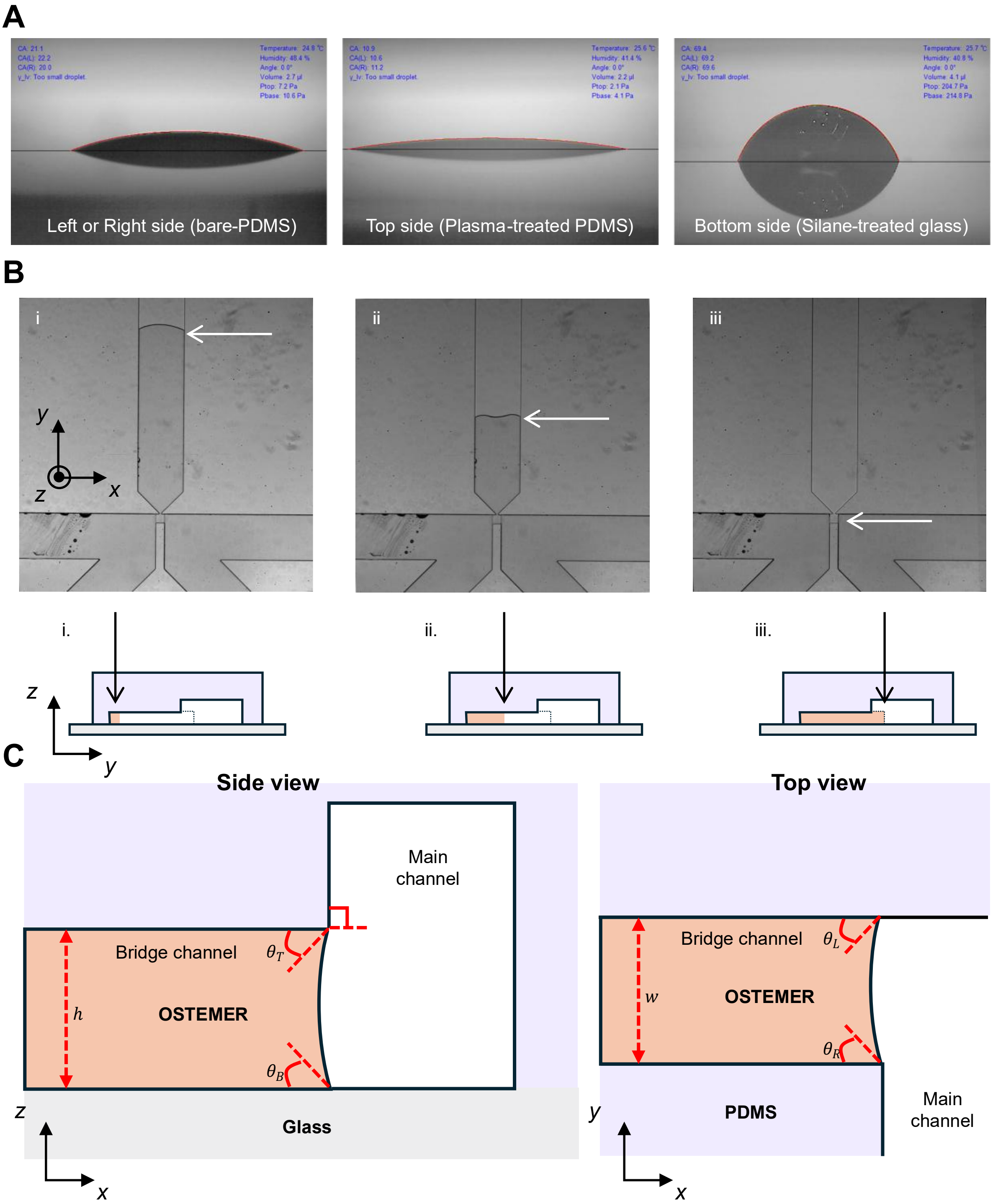

Figure S2. Wettability of various substrates and selective filling of OSTEMER resin into the bridge channel in the WNAD. (A) Contact angle (CA) measurements of the channel surfaces/walls. The left and right sides of the bridge channel correspond to bare PDMS, showing a CA of 21.1°. The top side (oxygen plasma-treated PDMS) exhibited a reduced CA of 10.9°, while the bottom side (silane-treated glass substrate) showed a high CA of 69.4°. (B) Sequential optical microscopy images showing the progression of OSTEMER resin from the injection channel into the bridge channel (i–iii). Cross-sectional illustrations below each image depict resin progression across the confined geometry. (C) Schematic illustrations of the cross-sectional (left) and top (right) views of the bridge channel. The diagrams indicate the channel

height (h), width (w), and contact angles (CAs) at the top (θ_T), bottom (θ_B), left (θ_L), and right (θ_R), which collectively determine the resin confinement by the capillary effects.

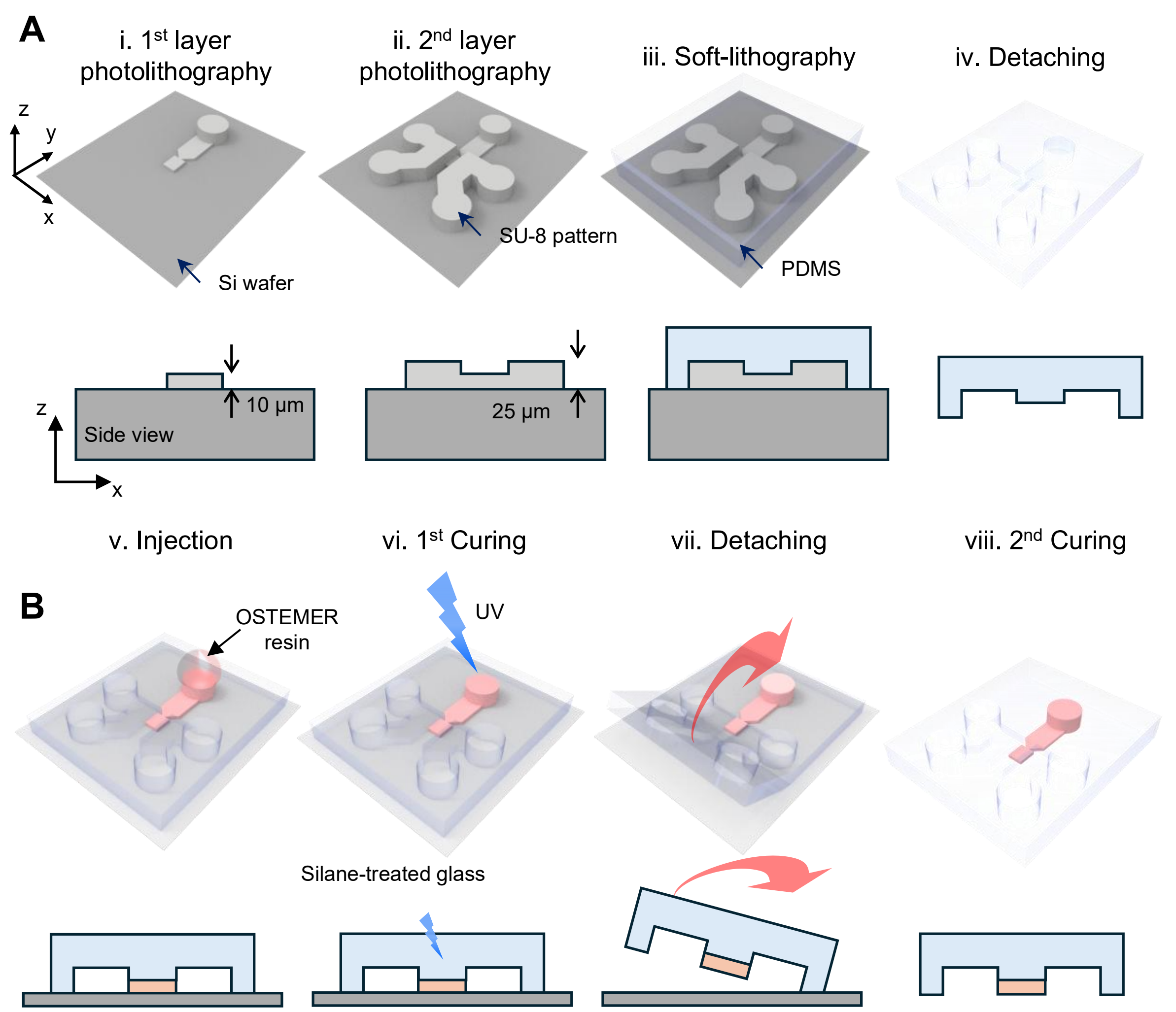

Figure S3. Fabrication process of a PDMS/OSTEMER hybrid device. (A) Photolithography process for fabricating a microfluidic device with two different height levels. (i) A single-layer photolithography pattern was fabricated with a thickness of 10 µm to define the injection and bridge channel. (ii) A second photolithography layer with a thickness of 25 µm was then patterned to form the main channel structure. (iii) PDMS was cast against the silicon mold containing both the 10 µm and 25 µm features to replicate the designed channel geometry. (iv) After curing, the PDMS replica was peeled off from the silicon mold to obtain the microchannel device. (B) Fabrication process for the PDMS/OSTEMER hybrid device. (v) The PDMS device was bonded to a silane-treated glass substrate following mild oxygen plasma treatment, and OSTEMER resin was injected into the inlet of the bridge channel. The resin selectively filled the injection and bridge channels due to the capillary effect caused by the shallow channel height (10 µm), while remaining excluded from the main channel (25 µm). (vi) The injected OSTEMER resin was cured under UV illumination to solidify the structure. (vii) After curing, the PDMS/OSTEMER hybrid device was carefully detached from the silane-treated glass substrate. (viii) The completed PDMS/OSTEMER hybrid device is shown, which integrates PDMS microchannels with selectively filled OSTEMER regions for subsequent bonding to the wrinkle substrate.

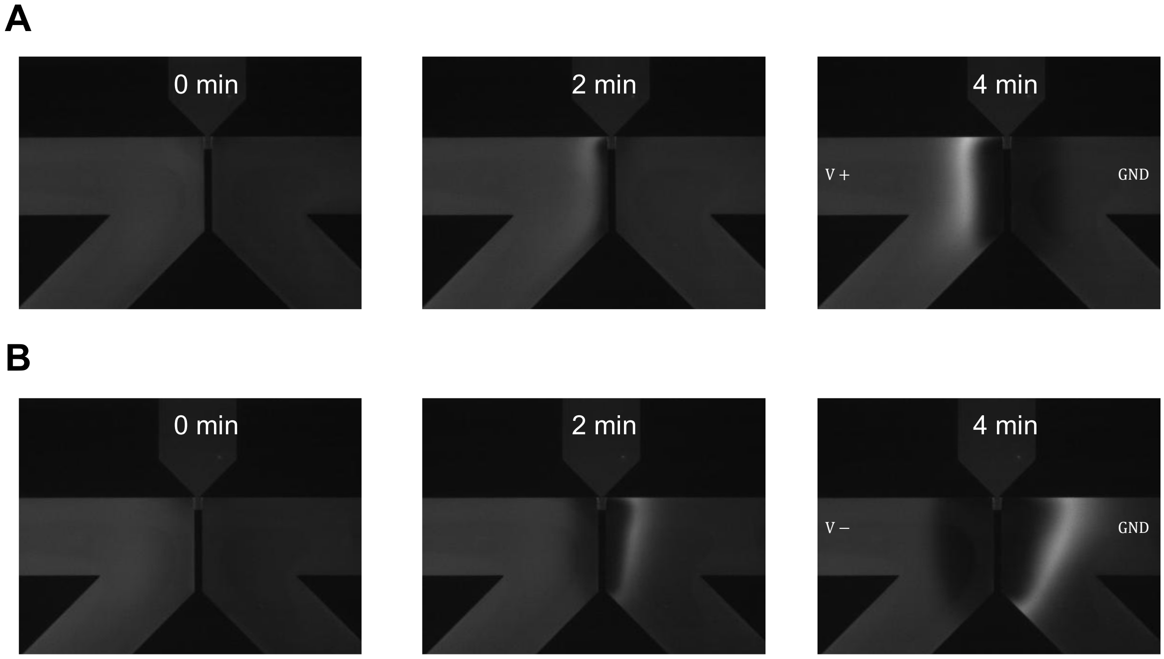

Figure S4. Time-lapse fluorescence imaging of ICP in wrinkle-based nanochannels. (A) Fluorescence images acquired at $t = 0$, 2, and 4 min under a forward bias of 3 V, showing FITC accumulation on the left side and depletion on the right side of the nanochannels. (B) Corresponding images obtained under a reverse bias of -3 V at the same time points, showing FITC accumulation on the right side and depletion on the left side of the nanochannels.

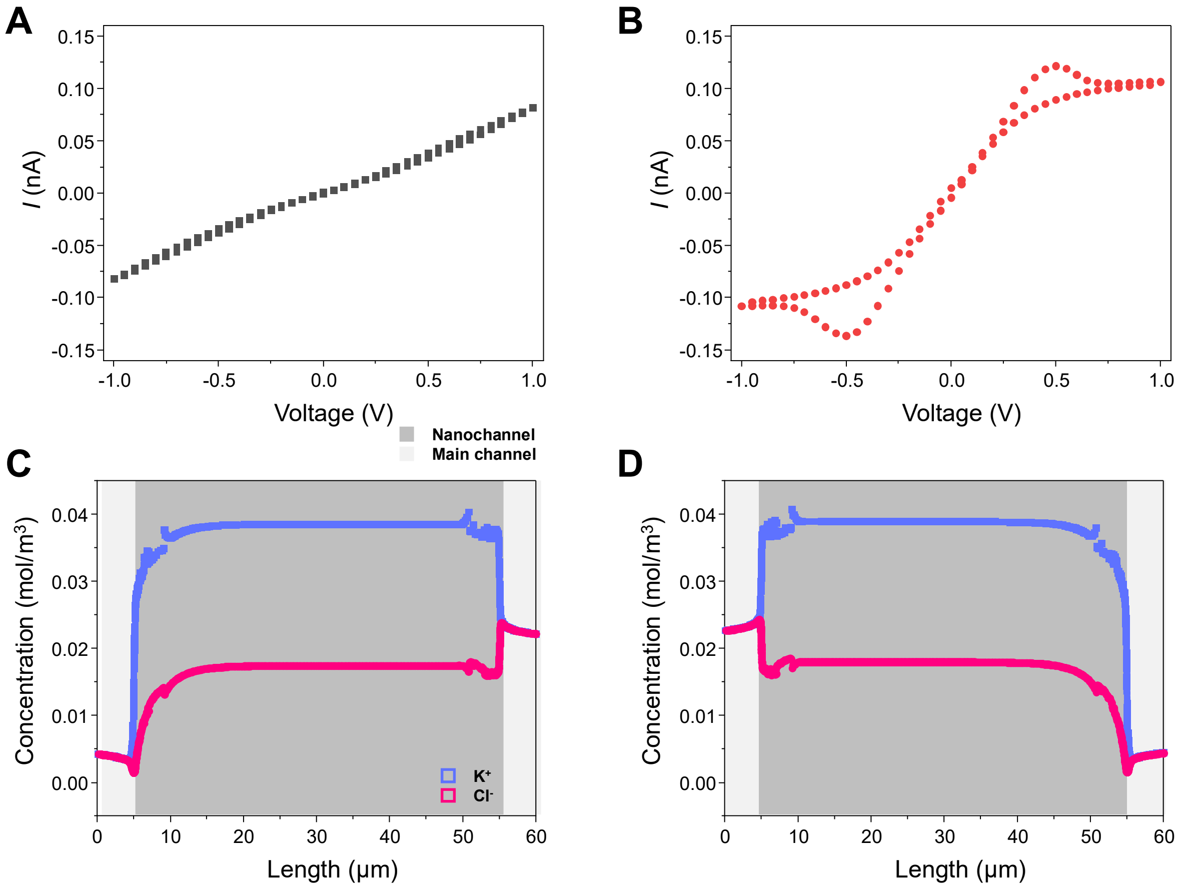

Figure S5. Numerical simulation results of ion transport and hysteresis behavior. (A) Simulated current-voltage (I-V) curve of a single wrinkle-based nanochannel (1-NC) under 10 μM KCl, showing an almost linear response with weak nonlinearity and negligible hysteresis, indicating limited ion polarization within a single transport pathway. (B) I-V curve of five parallel wrinkle-based nanochannels (5-NCs), exhibiting pronounced nonlinearity and a distinct hysteresis loop arising from enhanced ICP and interchannel coupling. (C) Spatial distributions of K^+ and Cl^- concentrations across the nanochannel including the left and right microchannels at +1 V bias. Ion depletion is observed at the biased left microchannel, while accumulation occurs at the grounded right side. (D) Ion concentration profiles under -1 V bias showing reversed behavior, with accumulation on the left and depletion on the right microchannel. Due to the cation-selective nature of the wrinkle-based nanochannel, the K^+ concentration inside the nanochannel remains higher than that of Cl^- under both bias conditions.

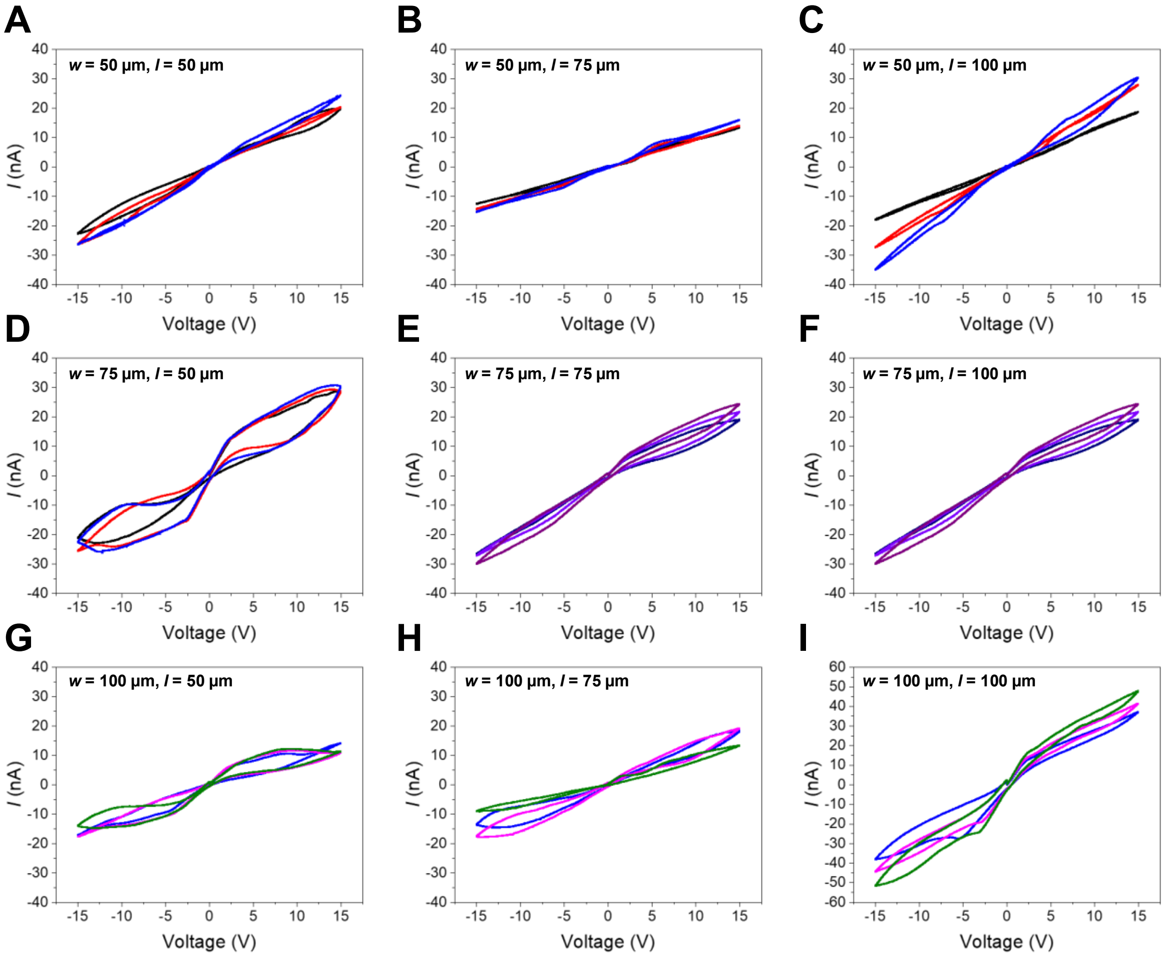

Figure S6. Hysteresis loops in I-V curves of the WNAD under different bridge channel geometrical dimensions. (A–I) I-V curves for the WNAD with various nanochannel geometries which correspond to combinations of bridge width w = 50, 75, and 100 µm and nanochannel length l = 50, 75, and 100 µm, respectively. Each measurement was repeated three times and averaged.

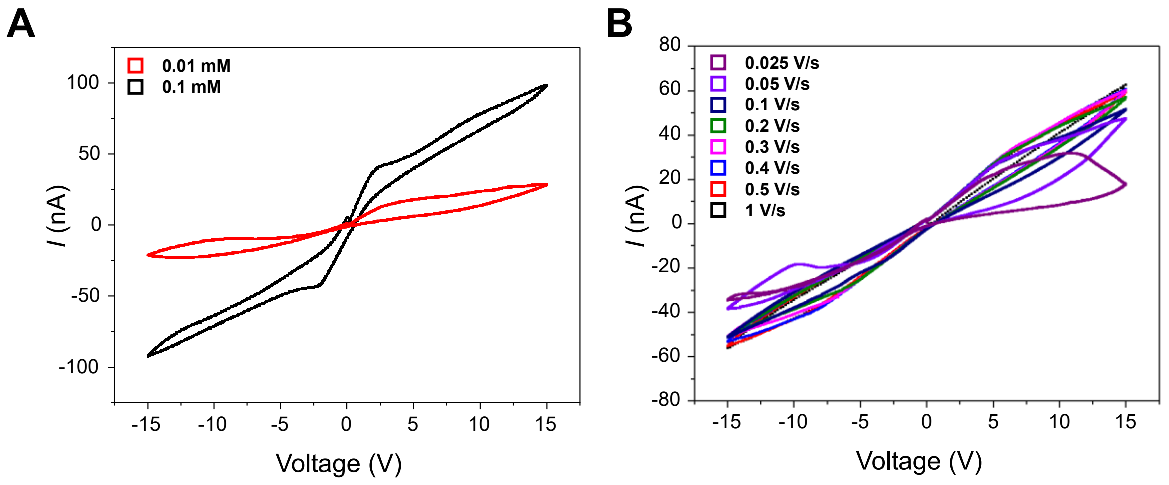

Figure S7. Effect of electrolyte concentration and scan rate on the hysteresis loop area. (A) Hysteresis loop area as a function of electrolyte concentration for the WNAD with $w = 75$ μm and $l = 50$ μm. (B) Hysteresis loop area as a function of voltage scan rate ranging from 0.025 V/s to 1 V/s.

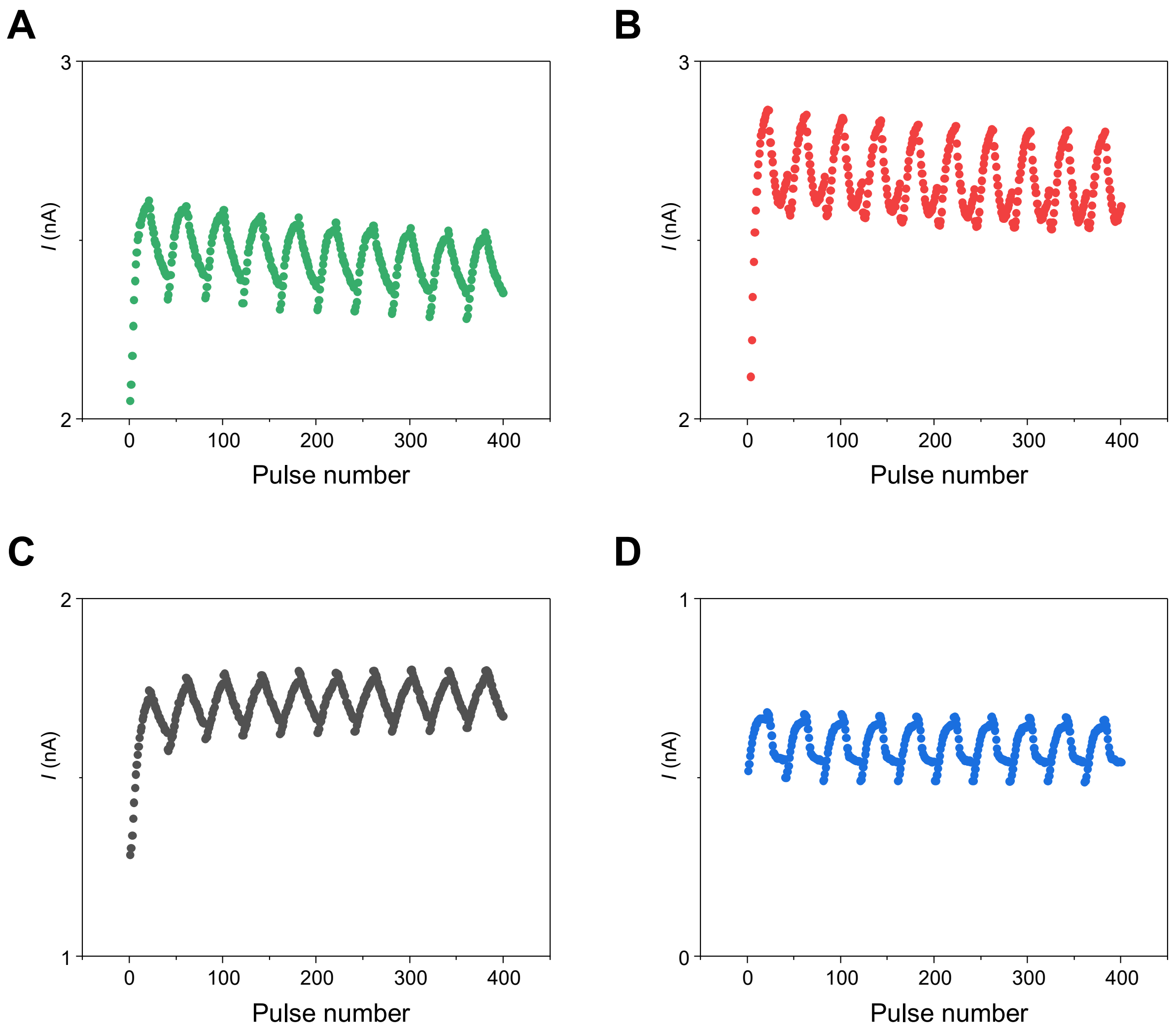

Figure S8. Potentiation-depression cycling of the WNAD under different geometrical dimensions. (A) Current measurement for w = 50 µm and l = 50 µm. (B) Current measurement for w = 100 µm and l = 50 µm. (C) Current measurement for w = 75 µm and l = 75 µm. (D) Current measurement for w = 75 µm and l = 100 µm. Potentiation and depression cycles were repeated 10 times for devices with varying the bridge channel dimensions. Write pulses of ±3 V (100 ms duration, 100 ms interval) were applied, while read pulses were fixed at +1 V (100 ms duration, 100 ms interval).

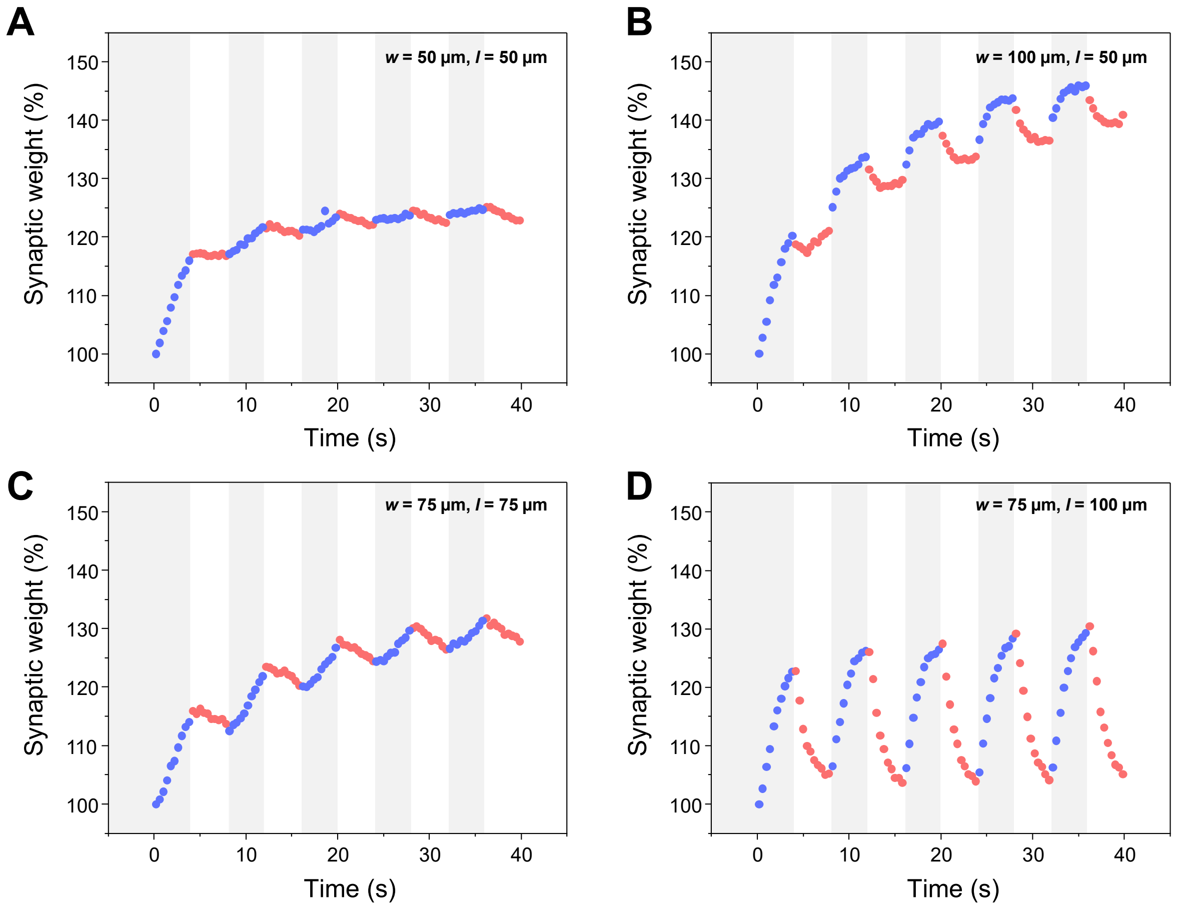

Figure S9. Conductance evolution of the WNAD with different geometrical dimensions. (A) Synaptic weight for *w* = 50 μm and *l* = 50 μm. (B) Synaptic weight for *w* = 100 μm and *l* = 50 μm. (C) Synaptic weight for *w* = 75 μm and *l* = 75 μm. (D) Synaptic weight for *w* = 75 μm and *l* = 100 μm. Synaptic weight changes were monitored by applying 10 write pulses (blue dots, +3 V, 100 ms duration, 100 ms interval) followed by 10 read pulses (red dots, +1 V, 100 ms duration, 100 ms interval).

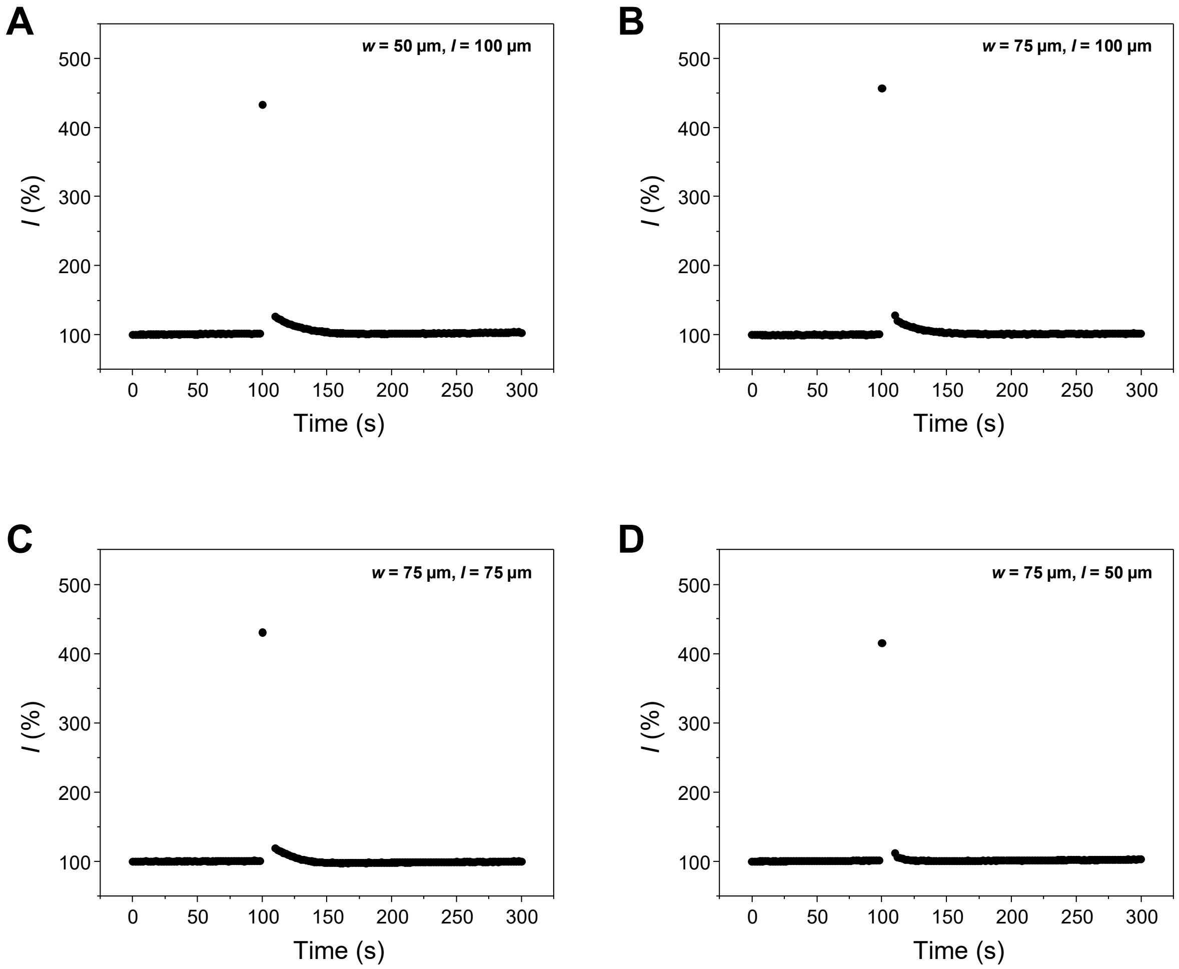

Figure S10. Geometry-dependent decay characteristics. (A–D) I-V responses showing conductance decay after a write operation for devices with different nanochannel geometries.

– *End of Document* –
